# Tuning Properties of Iron Oxide Nanoparticles in Aqueous Synthesis without Ligands to Improve MRI Relaxivity and *SAR*

**DOI:** 10.3390/nano7080225

**Published:** 2017-08-18

**Authors:** Debora Bonvin, Duncan T. L. Alexander, Angel Millán, Rafael Piñol, Beatriz Sanz, Gerardo F. Goya, Abelardo Martínez, Jessica A. M. Bastiaansen, Matthias Stuber, Kurt J. Schenk, Heinrich Hofmann, Marijana Mionić Ebersold

**Affiliations:** 1 Powder Technology Laboratory, Institute of Matesrials, Ecole Polytechnique Fédérale de Lausanne (EPFL), 1015 Lausanne, Switzerland; debora.bonvin@epfl.ch (D.B.); heinrich.hofmann@epfl.ch (H.H.); 2 Interdisciplinary Centre for Electron Microscopy (CIME), Ecole Polytechnique Fédérale de Lausanne (EPFL), 1015 Lausanne, Switzerland; duncan.alexander@epfl.ch; 3 Instituto de Ciencia de Materiales de Aragón, Universidad de Zaragoza, C/Pedro Cerbuna 10, 50009 Zaragoza, Spain; amillan@unizar.es (A.M.); pinol@unizar.es (R.P.); 4 Instituto de Nanociencia de Aragón, Universidad de Zaragoza, Mariano Esquillor s/n, 50018 Zaragoza, Spain; beasanz@unizar.es (B.S.); goya@unizar.es (G.F.G.); 5 Grupo de Electrónica de Potencia y Microelectrónica, I3A, Universidad de Zaragoza, 50018 Zaragoza, Spain; amiturbe@unizar.es; 6 Department of Radiology, University Hospital (CHUV) and University of Lausanne (UNIL), 1011 Lausanne, Switzerland; jbastiaansen.mri@gmail.com (J.A.M.B.); Matthias.Stuber@chuv.ch (M.S.); 7 Center for Biomedical Imaging (CIBM), 1011 Lausanne, Switzerland; 8 CCC-IPSB, Ecole Polytechnique Fédérale de Lausanne (EPFL), 1015 Lausanne, Switzerland; kurt.schenk@epfl.ch

**Keywords:** iron oxide nanoparticles, magnetic nanoparticle, aqueous synthesis, hydrothermal treatment, saturation magnetization, MRI relaxivity, specific absorption rate

## Abstract

Aqueous synthesis without ligands of iron oxide nanoparticles (IONPs) with exceptional properties still remains an open issue, because of the challenge to control simultaneously numerous properties of the IONPs in these rigorous settings. To solve this, it is necessary to correlate the synthesis process with their properties, but this correlation is until now not well understood. Here, we study and correlate the structure, crystallinity, morphology, as well as magnetic, relaxometric and heating properties of IONPs obtained for different durations of the hydrothermal treatment that correspond to the different growth stages of IONPs upon initial co-precipitation in aqueous environment without ligands. We find that their properties were different for IONPs with comparable diameters. Specifically, by controlling the growth of IONPs from primary to secondary particles firstly by colloidal and then also by magnetic interactions, we control their crystallinity from monocrystalline to polycrystalline IONPs, respectively. Surface energy minimization in the aqueous environment along with low temperature treatment is used to favor nearly defect-free IONPs featuring superior properties, such as high saturation magnetization, magnetic volume, surface crystallinity, the transversal magnetic resonance imaging (MRI) relaxivity (up to *r*_2_ = 1189 mM^−1^·s^−1^ and *r*_2_/*r*_1_ = 195) and specific absorption rate, *SAR* (up to 1225.1 W·g_Fe_^−1^).

## 1. Introduction

Iron oxide nanoparticles (IONPs) are widely used for numerous applications from solid state [1,2] to biomedical ones [3,4,5,6,7,8,9,10,11,12]. For all these applications, one needs to control simultaneously numerous mutually related properties of IONPs, such as composition, structure, morphology, crystallinity, magnetic volume, saturation magnetization etc. Moreover, all these IONPs’ properties either directly or indirectly determine specific application-related properties, such as magnetic resonance imaging (MRI) relaxivity (*r*) or specific absorption rate (*SAR*) related to biomedical applications as MRI contrast agents or as mediators for the hyperthermia treatment, respectively. Furthermore, fine-tuning of the IONPs’ properties has been shown to strongly influence both *r* and *SAR* due to their complex dependence on IONPs’ properties, which are in addition mutually related in a non-linear manner. In fact, it has been shown that some of the small changes in one IONPs’ property could dramatically affect even a few other properties. For instance, the magnetic volume is typically smaller than the IONPs’ volume due to a “magnetically dead” surface layer of disordered spins. This is often considered to be a consequence of structurally disordered (i.e., non-crystalline) surfaces around the crystalline IONPs. Thus, high surface crystallinity is needed to increase the magnetic volume via minimizing the thickness of the spin-disordered or magnetically dead surface layer. Besides the crystallinity of IONPs, the shape of IONPs also affects spin ordering at their surface. Namely, specific shapes are preferred for specific compositions, i.e., crystalline structures. For example, for IONPs with a cubic crystal structure, i.e., Fe_3_O_4_ and γ-Fe_2_O_3_, a cubic shape is preferred over the spherical one, because surface spins of a sphere are highly canted against external magnetic fields on most of the surface, while the surface spin state in cubes has close similarity with the core spin state [13]. Indeed, numerous studies of IONPs with cubic shapes reported advantageous properties as compared to IONPs with a similar size but non-cubic shapes, such as higher values of saturation magnetization [14,15,16,17], which is directly related to the magnetic volume and thus, to the thickness of the magnetically dead surface layer. Consequently, IONPs with controlled shapes have been reported to have among the highest *r* and *SAR* values for IONPs: *r*_2_ relaxivity of 761 mM^−1^·s^−1^ at 3 T for cubes of 22 nm [15] and *SAR* of 2560 W·g_Fe_^−1^ at 20.7 kA·m^−1^ field strength and 325 kHz for octahedrons of 40 nm [18]. Beyond the shape, the dipolar interaction strongly affects both *r*_2_ and *SAR*. *r*_2_ of 835 mM^−1^·s^−1^ (*r*_2_/*r*_1_ = 139) at 3 T was reported for mesoscopic IONP clusters [19] and 675 mM^−1^·s^−1^ at 3 T was reported for worm-like IONP clusters [20], while *SAR* of 960 W·g^−1^ at 410 kHz and field amplitude 10 kA·m^−1^ was reported for bacterial magnetosomes with a mean core diameter of about 30 nm [21]. Thus, the simultaneous control of numerous IONPs’ properties is essential to obtain high *r*_2_ and *SAR*. Such a control of the IONPs’ morphology, magnetic volume and other properties is difficult, but it can be achieved typically by the specific combination of organic solvent(s) and/or ligand(s) with other optimal synthesis parameters (e.g., high temperatures, special atmosphere) [15]. However, numerous efforts have been recently put in green environmental-friendly chemistry, and thus, in designing and studying chemical processes, which yield products with as-better-as-possible properties and which do not use organic solvents, special atmosphere, extreme conditions, ligands etc. [22,23]. Such a synthesis of IONPs with controlled properties, which result in *r*_2_ and *SAR* values comparable with values obtained by non-aqueous synthesis with ligands remains the challenge. To achieve this, one firstly need to understand the IONPs’ growth and changes under such conditions in a way, which allows to obtain at least the desired chemical composition and structure and to study the other properties in relation to the synthesis parameter(s). Therefore, our goal was to tackle these issues and to obtain IONPs with controlled properties under such conditions, and to correlate such a synthesis process with the properties of IONPs.

In order to control properties of IONPs in an aqueous synthesis and to separate nucleation and growth, we have developed a novel synthesis route which combines the co-precipitation (CP) and the hydrothermal treatment (HT), termed CP + HT [24]. In our previous study, we optimized some of the general synthesis parameters [24], and we found the optimal temperature for the HT step, which allowed high vacancy ordering in IONPs of γ-Fe_2_O_3_ studied by the synchrotron radiation powder diffractometry [25]. However, it is known that besides the temperature of the HT step, its duration has a crucial influence on the final synthesis product and therefore, that influence has to be studied into details. Since we previously optimized the other parameters, we here studied the influence of the HT treatment duration, which represents different growth stages of IONPs, on the size, crystallinity, structure, morphology, magnetic properties, MRI relaxivity and specific absorption rate of the obtained IONPs. The results showed an optimal duration of the HT treatment, which corresponds to IONPs with excellent properties.

## 2. Results

### 2.1. Synthesis, Structure and Crystallinity of IONPs

**Figure 1:**
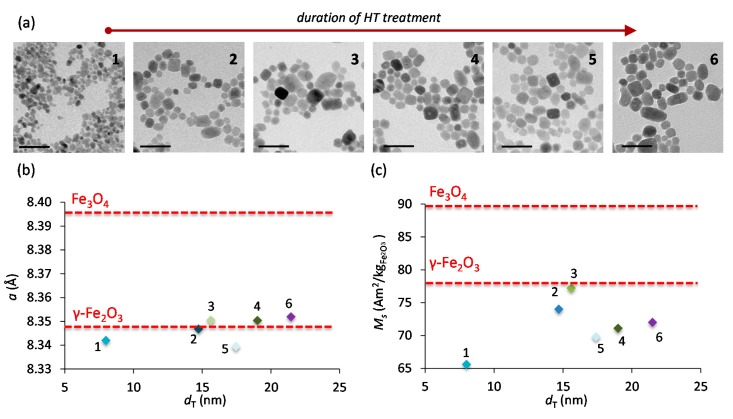
(**a**) Representative TEM micrographs of IONP samples. The scale bars on the micrographs are 50 nm. The red arrow on the top indicates the direction of the increase of the duration of the HT treatment. (**b**) Lattice parameter, and (**c**) saturation magnetization measured at 300 K for IONPs compared with corresponding values of stoichiometric maghemite (γ-Fe_2_O_3_) and magnetite (Fe_3_O_4_) used as reference and marked by red lines.

IONPs were synthesized by the CP + HT route, which consists of alkaline co-precipitation that results in instantaneous nucleation [26], and subsequent HT treatment, which allows the growth of previously formed nuclei. Briefly, the suspensions obtained by CP of ferrous and ferric chlorides in alkaline milieu were heat treated in autoclaves at 120 °C. To assess and follow the “evolution” of IONPs’ properties, the HT treatment was performed for different durations: 0, 6, 12, 15, 18 and 24 h; the corresponding sample names are 1–6, respectively. The obtained magnetite (Fe_3_O_4_) nanoparticles (NPs) were subsequently oxidized, yielding maghemite (γ-Fe_2_O_3_) IONPs, which were then characterized without size separation. Figure 1a shows representative transmission electron microscopy (TEM) micrographs of the obtained IONPs for different durations of the HT treatment. From manually measured Feret diameters of 500 IONPs’ in representative TEM micrographs, we calculated the equivalent diameter often termed “TEM diameter”, *d*_T_ (see the Experimental Section for calculation details, Figure S1 for additional TEM micrographs, and Figure S2 for *d*_T_ distribution). As expected, longer HT treatments resulted in larger particles (see Table 1).

The crystalline structure of IONPs was analyzed by X-ray diffraction, XRD (see patterns in Figure S3). All samples displayed the characteristic spinel structure (space group $Fd\bar{3}m$, number 227), which corresponds to both γ-Fe_2_O_3_ and Fe_3_O_4_. In fact, magnetite has larger values for all desirable properties than maghemite (e.g., saturation magnetization, see Figure 1c); however the latter is preferred for some applications, like for example for injectable nanomedicine, because the presence of Fe^2+^ in the former was previously reported to promote oxidative stress [27]. The distinction between these two iron oxides is possible by comparing the lattice parameters of the studied IONPs with those of stoichiometric γ-Fe_2_O_3_ (8.346 Å, JCPDS file 39-1346) and Fe_3_O_4_ (8.396 Å, JCPDS file 19-629). The lattice parameters of IONPs (Figure 1b and Table 1), as obtained by the refinement of XRD patterns match that of γ-Fe_2_O_3_ to within the standard deviation. The crystallite diameter of IONPs, *d*_C_ (Table 1), was obtained from XRD refinement combined with the Debye-Scherrer method using either only the narrowest line (404), or all lines of the eight Bragg reflections (for *d*_C_ calculated from all 8 lines separately see Table S1). The thereby obtained values of *d*_C_ agree with the *d*_T_ The typical peak broadening with decreasing IONPs’ size (especially in sample 1) is also observed, which also suggests the high defect density within IONPs in sample 1. As the duration of the HT treatment increases, both *d*_T_ and *d*_C_ increase correspondingly: both *d*_T_ and *d*_C_ doubled in sample 2 as compared to sample 1, while the following samples (3, 4 and 5) did not show any notable difference with sample 2. Further enlargement of *d*_T_ and *d*_C_ occurs again in sample 6.

The infrared (IR) spectra of IONPs (Figure S4) showed multiple bands between 800 and 400 cm^−1^, which suggests a structure of γ-Fe_2_O_3_ with ordered vacancies [28,29]. Also, by X-ray photoelectron spectroscopy (XPS) obtained core-level spectra of the Fe 2p displayed a satellite peak, which is characteristic for γ-Fe_2_O_3_ (Figure S5) [30].

Structural order at the surface of IONPs is crucial for numerous properties related to the magnetic volume, *V*_m_. Namely, the structurally disordered surface of magnetic NPs has also disordered spins. Consequently, the surface layer is magnetically inactive (“dead”) and the *V*_m_ is lower than volume, *V*, of NPs. This directly affects the saturation magnetization, *M*_s_ [28,29,31], which is thus proportionally lower than *M*_s_ of bulk, *M*_sb_. Therefore, we measured the magnetization as a function of the magnetic field strength (Figure S6) to obtain *M*_s_ values which were found to be below *M*_sb_ of maghemite (Figure 1c and Table S2). Comparing the obtained *M*_s_ values with *M*_sb_ of maghemite (about 78 A·m^2^·kg^−1^) allowed us to estimate the thickness of the magnetically dead layer, *t*, ranging from 0.3 to 3.2 Å. From this, we calculated *V*_m_ to be from 84 to 99% of *V*, meaning that the non-magnetic volume ranges from 1 to 16% of *V*; for values of *t* and *V*_m_/*V* see Table S3. It has to be pointed out that the obtained *t* values correspond to the approximate length of one to few unit cells. That also suggests that IONPs with low *t* have a highly crystalline surface.

**Figure 2:**
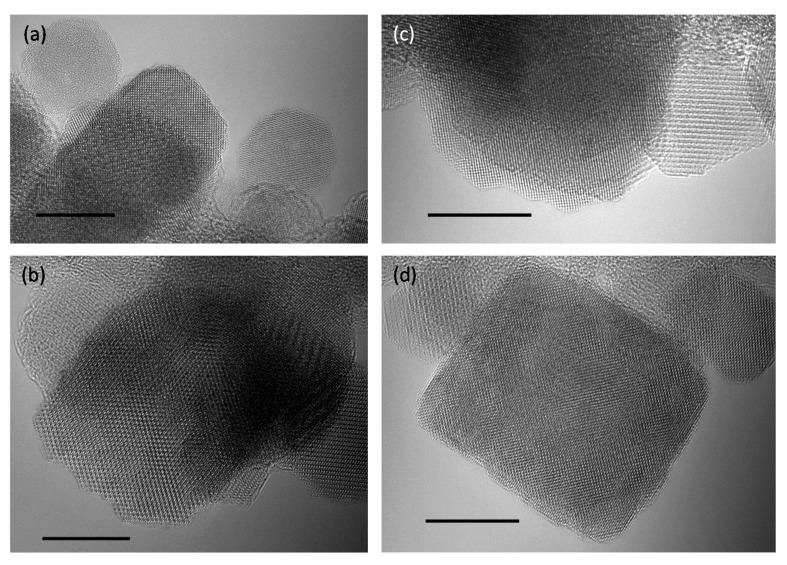
Aberration-corrected TEM micrographs of nearly defect-free highly crystalline surface of IONPs’ samples 4 (**a**,**b**) and 6 (**c**,**d**). All scale bars are 10 nm.

To verify this, we studied the IONP surface crystallinity using spherical aberration-corrected TEM with the effects of chromatic aberration reduced by monochromating the incident beam. With this state-of-the-art TEM imaging, possible crystalline disorder at the surface can be assessed in detail. Figure 2 shows that samples 4 and 6 have highly ordered crystalline surfaces. Note that these samples were structurally stable under the low energy 80 keV electron beam, except for apparent hopping of atoms at the edges of steps and kinks in monocrystalline IONPs (see Supplementary Video S1). Therefore, this high surface crystallinity is not the result of electron-beam induced crystallization of an initially disordered surface state. It is noted that high beam energies can instead strongly affect the structure, morphology and bonding of nanoparticle samples [32,33].

The high surface crystallinity of our IONPs can be explained by the aqueous environment during the synthesis. Specifically, the high polarity of the medium creates a high energy barrier which drives the recrystallization of IONPs to expose low energy surfaces. This process is enabled by direct interface of the surface of IONPs with water molecules without ligands, which would suppress the mass transport at the surface and lower the surface energy. Therefore, in an aqueous environment, the absence of ligands promotes the surface structural ordering and thus, high crystallinity in two ways: by creating a highly energetic interface with water and by allowing the surface atoms to freely rearrange without capping boundaries. In fact, highly crystalline magnetic NPs were typically reported in HT synthesis under harsh conditions (elevated temperatures and/or pressures), which were the cause for the obtained high crystallinity [34]. Importantly, these reported syntheses usually started by lowering the surface energy with suitable organic solvent(s) and/or ligand(s), and then by exposing IONPs to harsh conditions [34]. On the contrary, we started without ligand(s) from water as a solvent creating a high interfacial energy, and performed mild HT treatments. Our strategy is shown to be as successful by the obtained results.

### 2.2. Magnetic Properties

The hysteresis curves of IONP suspensions were measured at 250 and 300 K, i.e., in the frozen and liquid states, respectively (Figure S7). Values of coercive field *H*_c_ and remanent magnetization *M*_r_, extracted from these curves, are given in Table S2. IONPs did not show any significant coercivity in liquid suspensions (at 300 K), because even if some individual IONPs in these samples are in the ferrimagnetic state, they are free to rotate in liquid. However, at 250 K the solvent is frozen and hence, IONPs are in fixed positions without the possibility for Brownian rotation. In that case, IONPs at 250 K have *M*_r_/*M*_s_ ratio of up to 0.3 (see Table S2). That clearly shows a system of interacting IONPs since this ratio is 0.5 for non-interacting randomly oriented MNPs with uniaxial symmetry [35]. This is expected for IONP suspensions, which have higher concentrations (as in our samples, ~10 mg_Fe_·mL^−1^. It is common that values of *H*_c_ are used for the calculation of effective anisotropy constant (*K*_eff_), the effective anisotropy constant, according to the expression:

(1) $$H_{C}=\frac{2K}{M_{s}}\left[1-{\left(\frac{25k_{B}T}{KV_{m}}\right)}^{1/2}\right],$$

where *k_B_* is the Boltzmann constant, *V*_m_ is the magnetic volume, *T* is the temperature, and *M*_s_ is the saturation magnetization. We emphasize that this expression was derived for single domain non-interacting MNPs in superparamagnetic regime below the blocking temperature [36]. Therefore, as for instance in this case, applying it to interacting MNPs would result in incorrect *K*_eff_ values. Specifically, it results in overestimation of *K*_eff_ (as can be seen in Table S2), because the estimated values are enhanced by the contribution of dipolar interactions, which are rarely taken into consideration [35,37].

### 2.3. Morphology

By using state-of-the-art TEM imaging, differences in morphology were assessed; in sample: 1—small spherical IONPs; 2—“peanut shaped” IONPs resembling two small spherical IONPs from sample 1; 3 and 4—more rectangular; 5—again deformed; and 6—polycrystalline and clearly irregular in shape. Based on the above given physical/chemical properties, this morphological sequence can be correlated with the current knowledge of IONPs’ growth.

**Figure 3:**
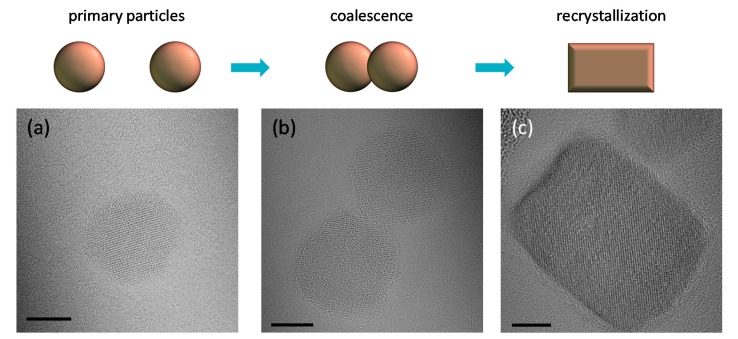
TEM micrographs and the corresponding scheme of growth from spherical primary particles (PPs) in sample 1 (**a**) by coalescence of PPs into secondary particles (SPs) in sample 2 (**b**) and their further recrystallization in sample 3 until rectangular and cubic shapes in sample 4 (**c**). All scale bars are 5 nm.

At the beginning of the synthesis, instantaneous seed formation occurs due to the highly alkaline environment (pH > 10) [26], yielding approximately spherical primary particles, PP, as seen in sample 1 (Figure 3a). An expected initial coalescence of such spherical PPs can be attributed to the “peanut shape” IONPs in sample 2 (Figure 3b). This suggests that growth of PPs proceeded by their aggregation into assemblies called secondary particles, SP, controlled by diffusion and collision, where colliding PPs may re-align themselves into a more favorable energy state [38,39,40], such that adjacent PPs’ share a common crystallographic orientation across an interface [38]. This mechanism is favored by hydrophobic/hydrophilic interactions due to the different polarity of crystallite surfaces [41]; this is the driving force to self-assemble PPs in the mechanism known as “brick-by-brick” assembly [42]. In γ-Fe_2_O_3_, the typically exposed low energy surfaces are (100), (111) and (110) [43,44,45]. The latter is non-polar, meaning relatively hydrophobic, while the first two surfaces are the polar surfaces with exposed metal ions (i.e., more hydrophilic) and tend to reconstruct and become non-polar [43,44,45]. These largely abundant non-polar surfaces would have a higher surface energy, and would thus cause PPs’ coalescence in order to reduce the overall free energy [46]. Therefore, such merging of typically two PPs would give SPs as seen in sample 3 with the same “width” but double the “length” of the PPs observed in sample 1. 

Typically, after coalescence, SPs recrystallize and indeed IONPs in samples 3 and 4 have more rectangular and square shapes (Figure 3c). These shapes are close to a (100) cubic morphology indicating more energetically favorable crystallographic faceting (Figure 2a,b and Figure 3c). Li et al. also observed changes of shape of IONPs from spherical to cubic in their enlargement with prolonged reflux time in HT treatment [47]. Moreover, for a cubic lattice, the spin states at surfaces of such shapes are expected to be closer to the core spin states than for spherical shapes [28,29,48]. Therefore, samples with more cubic IONPs would have lower *t* (i.e., higher *M*_s_), as for instance observed in sample 3, which has a *V*_m_ of 99% of *V*.

**Figure 4:**
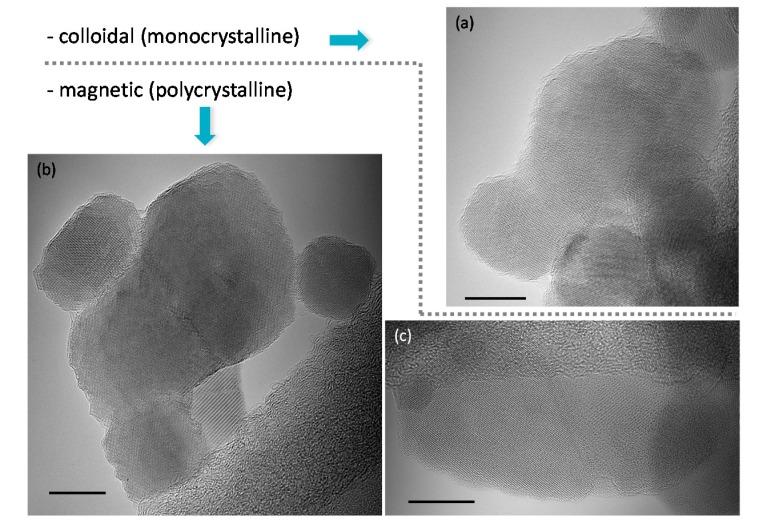
Representative TEM micrographs of monocrystalline secondary particles, SPs, (**a**) and polycrystalline SPs (**b**,**c**) separated by grey horizontal dashed line. The corresponding interaction (colloidal and magnetic interactions) and the type of SPs (monocrystalline and polycrystalline) caused by this interaction are indicated by the arrows. All scale bars are 10 nm.

For longer HT treatment, coalescence can continue by the above-described colloidal interactions, i.e., through a mechanism of oriented attachment of PPs, which gives monocrystalline SPs (Figure 4a). However, as the duration of the HT treatment increases, polycrystalline SPs were also observed, especially in sample 6 (Figure 4b,c). In these SPs, crystallographic planes are visibly mismatched at the grain boundaries (see Figure 4b,c and Supplementary videos 2 and 3). The monocrystalline grains correspond in size to the PPs and/or SPs of the first 4 samples (Figure 4b,c and Supplementary videos 2 and 3). With this lattice mismatch across boundaries, we suspect that these polycrystalline SPs were formed by magnetic interactions rather than colloidal ones. In fact, magnetic interactions *have* to be taken into account as soon as: (a) some individual MNPs are large enough to be ferrimagnetic (diameter larger than roughly about 26 nm in the case of γ-Fe_2_O_3_ IONPs) [49] or (b) suspensions of superparamagnetic IONPs with interparticle interactions. Actually, the *d*_T_ distribution of samples 2 to 6 (Figure S2) showed a fraction of IONPs larger than 26 nm (see Figure S2 for fraction’s values), which indicated the presence of ferrimagnetic IONPs (although their suspension behaves superparamagnetic as observed in the hysteresis curves, Figure S7). In fact, coalescence of two colliding IONPs could still yield monocrystalline SPs, if at least one colliding IONP re-aligns to match the crystallographic orientation at the joint interface leading to more favorable energy state. This would only be possible if at least one colliding IONPs is small (i.e., PP), because the frequency of rotation of IONPs is inversely proportional to their size [50]. On the contrary, the attractive magnetic interactions of colliding IONPs would give polycrystalline SPs, because the magnetic interactions would be prevalent over the colloidal ones and therefore the approaching IONP could not reorient to match the lattice. These all suggest that rather magnetic and colloidal interactions would be responsible for polycrystalline and monocrystalline SPs, respectively (Figure 4).

### 2.4. MRI Relaxivity

Given the high *M*_s_, *V*_m_ and surface crystallinity of our IONPs needed for high MRI relaxivity, we measured their longitudinal (*T*_1_) and transversal (*T*_2_) relaxation times in a clinical 3 T magnetic resonance (MR) scanner, as well as their relaxivities (*r*_1_ and *r*_2_), which are defined as the slope of the inverse of *T*_1_ and *T*_2_, respectively_,_ as a function of the concentration of the contrast agent (see the obtained values of *r*_2_, *r*_1_, and *r*_2_*/r*_1_ in Tables S4 and S5). The inverse of *T*, e.g., the relaxation rate *R*, linearly depends on the IONP concentration only for homogeneously dispersed particles; thus, only a limited range of IONP concentrations were considered: 0.5 to 20 µg_Fe_·mL^−1^. The hydrodynamic diameters, *d*_H_, of IONPs measured at similar concentrations as for the relaxivity measurements (see Table 1 and Figure S8) indicate that there are typically few IONPs per agglomerate as compared to the corresponding *d*_T_, which does not completely exclude the presence of magnetic interactions. For samples 2–6, the found *R*_1_ and *R*_2_ values were linearly dependent on the concentration (see Figure S9). Hence, a contribution of the interparticle interaction in the measured concentration range can be considered as negligible. Thus, differences in relaxivities between samples 2 to 6 with similar *d*_T_ can be approximated to originate from differences in the intrinsic natures of their IONPs.

**Figure 5:**
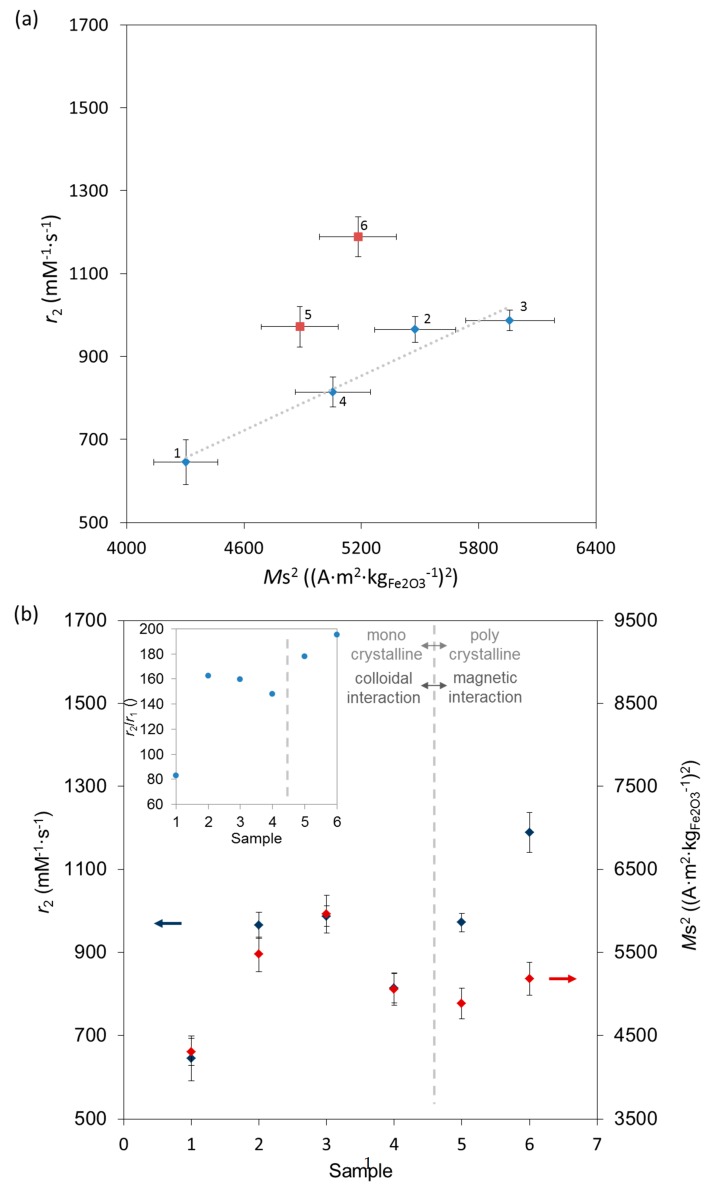
(**a**) MRI transverse relaxivity (*r*_2_) as a function of square of corresponding saturation magnetization (*M*_s_) shows increase except for the samples 5 and 6 (marked red); (**b**) *r*_2_ in blue and (*M*_s_)^2^ in red, given versus samples of IONPs along with the increased duration of the HT treatment. Insert shows the relaxivity ratio (*r*_2_*/r*_1_) versus samples.

Even though the relaxivities are a complex function of numerous parameters [34], the transverse relaxivity (*r*_2_) is proportional to *M*_s_^2^ [51,52]. This expected increase of *r*_2_ along with *M*_s_^2^ was observed only for the first 4 monocrystalline samples, while for polycrystalline samples 5 and 6 a discrepancy between the trends in *r*_2_ and *M*_s_^2^ can be noticed (Figure 5a,b). In fact, *M*_s_ cannot increase above the value in bulk γ-Fe_2_O_3_. In addition, an increase of the particle’s size (i.e., a decrease in surface area) [53] and/or clustering [54] strongly affects *r*_2_ as well as *r*_2_*/r*_1_, primarily due to the higher local magnetic moment and consequential dipolar interactions [34]. Therefore, we also plotted *r*_2_ and *M*_s_^2^ as a function of *d*_T_ and *d*_C_ (Figure S10); as observed above, *M*_s_^2^ follows the same trend as *r*_2_ for the first 4 samples. Moreover, *r*_2_ increases with increasing IONPs’ size in the first three samples, which is characteristic for the “motion averaging regime” (MAR) valid for relatively small homogeneously dispersed particles [53], but breaks down for larger particles [53], as we also see. Figure 5b further shows that samples with similar sizes, such as samples 2 to 5, have different *r*_2_ and *M*_s_^2^, which can be associated to the observed morphology changes between IONPs’ samples, as previously shown [16,55,56]. Namely, more rectangular and faceted IONPs in sample 3, which hence have the lowest *t*, i.e., the largest relative *V*_m_ (99% of *V*), i.e., the highest value of *M*_s_, have consequently one of the highest values of *r*_2_. It could be expected that *r*_2_ decreases for the large polycrystalline IONPs in sample 6 (which is out of the MAR regime), as observed by others [16]. Instead, the highest *r*_2_ value was found in sample 6. In fact, 24.6% of IONPs in this sample were above 26 nm (i.e., ferrimagnetic). This strongly suggests the presence of dipolar interactions known to result in an increase of *r*_2_ [34,54], which could explain the observed increase of *r*_2_ in sample 6.

Previous studies have found that an increase in the particle’s size (i.e., a decrease in surface area) is associated with a decrease in their *r_1_* values, which agrees with our results (see Figure S11); sample 1 with the largest specific surface area, *SSA*, of 170.33 m^2^·g^−1^ (see Table 1) has the largest *r*_1_. Among the other samples (2–6), sample 3 with rectangular faceted IONPs had one of the largest *r*_1_ values. This is in agreement with reports of Gao’s group [16,55] that *r*_1_ is larger for IONPs with metal-exposed surfaces such as (100) and (111), which are the typically-exposed low-energy surfaces in γ-Fe_2_O_3_.

Among the highest reported *r*_2_ values is 958 mM^−1^·s^−1^ found in cobalt ferrite (Co_0.5_Fe_2.5_O_4_ composition) at 0.5 T [56]. Among IONPs, the *r*_2_ relaxivity of: 835 mM^−1^·s^−1^ (*r*_2_/*r*_1_ = 139) at 3 T was reported for mesoscopic IONP clusters having significant dipolar interactions within the porous matrix [19]; 761 mM^−1^·s^−1^ at 3 T was reported for cubic IONPs of 22 nm [15]; 679.3 mM^−1^·s^−1^ at 7 T was reported for octapod IONPs [55]; 675 mM^−1^·s^−1^ at 3 T was reported for worm-like IONP clusters [20]; or 509 mM^−1^·s^−1^ at 7 T was reported for 16 nm cubic IONPs [57]. Otherwise among commercialized IONPs, the *r*_2_ relaxivity was 189 mM^−1^·s^−1^ (*r*_2_/*r*_1_ = 19.5) [58] for Resovist (Bayer Schering Pharma AG, Berlin, Germany, commercially-abandoned in 2009). Since T2-weighted applications require contrast agents with both high *r*_2_ and high *r*_2_/*r*_1_, our results (the *r*_2_ of up to 1189 mM^−1^·s^−1^ and *r*_2_/*r*_1_ of up to 195) show that our IONPs have properties beneficial for T2 contrast agents.

### 2.5. Specific Absorption Rate

Furthermore, we evaluated the heating potential of our IONPs by measuring *SAR*, i.e., the rate of heat dissipation per unit mass of MNPs, which is essentially determined by three material’s properties: Néel and Brownian relaxation times (related to spin and particle relaxation, respectively), and hysteresis losses. The highest *SAR* values were found for MNPs which are ferromagnetic and with hard magnetic phase or exchange coupling between phases [14,59,60,61]. However, for nanomedicine, superparamagnetic and soft magnetic phase MNPs are favored over ferrimagnetic and hard magnetic phase, since the latter foster thrombosis and agglomeration [62]. This preferred magnetic state was shown for our IONPs (see Figure S7 for *M*(*H*) curves, and Table S2 for *H*_c_ and *M*_r_ at 300 K). For our superparamagnetic IONPs’ suspension, we measured higher *SAR* values in water than in agar gels, in which IONPs are fixed and cannot rotate, confirming the expected contribution of the Brownian relaxation in addition to the Néel contribution (see Figure S12).

*SAR* values were obtained by using the formula:

(2) $$SAR=\left(\frac{C_{pH_{2}O}}{m_{\mathrm{Fe}2O3}}\right)\cdot \left(\frac{dT}{dt}\right),$$

where $m_{{\mathrm{Fe}}_{2}O_{3}}$ is the concentration of IONPs (in gram of γ-Fe_2_O_3_ per liter), $C_{pH_{2}O}$ is the specific heat capacity of water, and $\frac{dT}{dt}$ is the slope of the linear part of the heating curve (see the examples in Figures S12–S14). The increase in temperature with time (heating curve) was measured minimum three times, which showed good reproducibility (see Figure S13). The heating curves of one sample at different IONPs concentrations (see Figure S14) gave the same *SAR* values suggesting no strong interparticle interactions at the measured concentrations, as also seen above from the dependence of the relaxation rate on the IONP concentration.

**Figure 6:**
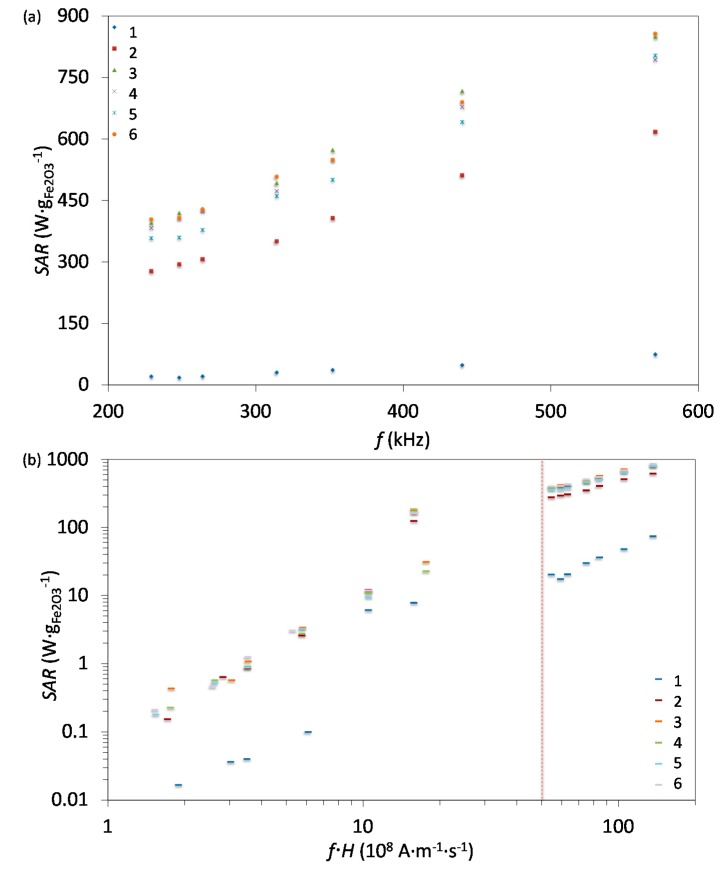
(**a**) *SAR* values as a function of frequency at the field amplitude of 23.9 kA·m^−1^ measured for all 6 samples. (**b**) *SAR* values as a function of the *f·H* product for measured values of *f* and *H*. The vertical dashed lines indicate two biological limits for the *f·H* product depending of the exposed volume, which corresponds to indicated hyperthermia coil size.

Since *SAR* depends on parameters of the alternating magnetic field, frequency *f* and amplitude *H*, we measured *SAR* at different values of *f* and *H*. For instance, Figure 6a shows *SAR* values measured at *H* of 23.9 kA·m^−1^ and at frequencies ranging from 200 to 600 kHz. As expected, *SAR* increased with increasing frequency, an increase that is more pronounced for larger IONPs, as also observed by others [63]. Besides the correlation of *SAR* with *f* and *H* individually, their product *f·H* is important for clinical applications and must not exceed 5 × 10^9^ A·m^−1^·s^−1^ for a safe clinical treatment by hyperthermia depending on the exposed volume [59]. Figure 6b shows *SAR* vs. the *f·H* product, where the vertical dashed line indicates the given clinical boundary for *f·H*. 

**Figure 7:**
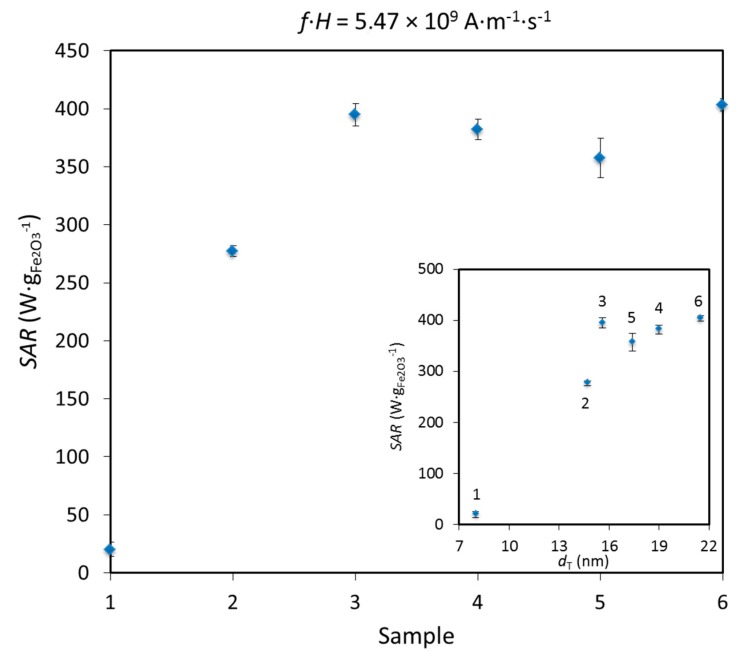
*SAR* as a function of the sample’s number to observe the evolution of *SAR* with the prolonged HT duration of IONPs at the field parameters the closest to the clinical limit of the *f·H* product. The insert shows *SAR* as a function of *d*_T_ at the same field parameters. The error bars indicate standard deviation.

The variation of *SAR* is typically plotted versus *d*_T_ (see insert in Figure 7). From the theory for superparamagnetic MNPs, for constant *f* and *H*, *SAR* should increase with the TEM size until a maximum, followed by a decrease [59,64,65]. We indeed observed this behavior for the first 5 samples, but *SAR* increased again in sample 6 (see Figure S15 for *SAR* vs. *d*_T_ at other *f·H* values). One would expect that an increase in diameter of IONPs coupled with the formation of grain boundaries in polycrystalline sample 6 would lower *SAR*, as already reported [66]. Instead, an increase in *SAR* of sample 6 can be explained by dipolar interactions, which were already observed for these polycrystalline IONPs and which are known to enhance *SAR* values [67,68]. We have to note that an abrupt jump in *SAR* values was observed between samples 1 and 2, as for *r*_2_. Taking into account the “peanut-shaped” morphology of IONPs in sample 2, these IONPs can be viewed as short magnetic chains consisting of minimum two units and a chain formation is known to increase *r*_2_ and *SAR* [67,69].

In order to compare *SAR* values measured at different fields, Pankhurst introduced the intrinsic loss power (*ILP*) as *SAR* normalized to *f·H*^2^ [70], which applies only to superparamagnetic MNPs in the Néel relaxation model, small field amplitudes, etc. [59,65,70]. While we report the *ILP* vs. *f·H* in Figure S16, with sample 6 giving the highest *ILP* value of 3.1 nH·m^2^·kg^−1^ (*SAR* = 403 W·g_Fe2O3_^−1^, *f·H* = 5.47 × 10^9^ A·m^−1^·s^−1^), we note that this model cannot be applied to our system because of its limited validity, which is often neglected, as stressed by Dutz and Hergt [46].

Outside of the clinically applicable conditions (at 571 kHz and 23.9 kA·m^−1^, *f·H* = 13.64 × 10^9^ A·m^−1^·s^−1^), *SAR* values of our samples were up to 856.7 W·g_Fe2O3_^−1^ (or 1225.1 W·g_Fe_^−1^). However, close to the clinically relevant conditions (at *f·H* = 5.47 × 10^9^ A·m^−1^·s^−1^), the measured *SAR* was up to 403 W·g_Fe2O3_^−1^ (or 576.3 W·g_Fe_^−1^). In comparison, among numerous other previous studies of *SAR* of engineered IONPs at *f·H* close to the clinical limit (~5 × 10^9^ A·m^−1^ s^−1^), *SAR* of 2560 W·g_Fe_^−1^ has been reported for octahedral IONPs of 40 nm [18], while *SAR* of ~1000 W·g_Fe_^−1^ has been reported for 27 nm IONPs, both synthesized by a non-aqueous route [71]. Otherwise, among natural products, Alphandéry has reported *SAR* of 875 W·g_Fe_^−1^ at comparable *f·H* (5.86 × 10^9^ A·m^−1^·s^−1^) for magnetosomes obtained from AMB-1 magnetotactic bacteria [72]. Nevertheless, previous *SAR* values for engineered IONPs synthesized by an aqueous route are far below these values.

## 3. Materials and Methods

### 3.1. Synthesis of Iron Oxide Nanoparticles (IONPs)

IONPs were synthesized by our novel CP + HT method with previously optimized part of synthesis parameters [24]. Briefly, aqueous solutions of FeCl_3_·6H_2_O and FeCl_2_·4H_2_O were prepared at room temperature with the relative fraction of Fe(II) in total Fe amount (*R*) of 0.5. Afterwards, 6 M Ammonia solution was mixed instantaneously with the solution of iron salts under vigorous stirring resulting in the immediate CP process. Note that this CP step was not performed under inert atmosphere, and thus, fast mixing without air bubbles was essential in order to avoid oxidation of Fe(II) before co-precipitation happens. The obtained suspension was instantaneously transferred into a sealed autoclave for the HT treatment (without stirring, since the agitation during aging broadens the size distribution) [73] at 120 °C for different durations: 0, 6, 12, 15, 18 and 24 h; corresponding sample names are 1–6, respectively. The resulting particles were washed from ammonia several times with double distilled water (ddH_2_O) until pH 7.5, centrifuged (Beckman Coulter; Avanti J-26 XP; Nyon, Switzerland, 5 min, 5000 RCF) in order to remove possible remaining molecules of ammonia and resuspended in 25.7 mL of 0.35 M Fe(NO_3_)_3_ and 17.4 mL of 2 M HNO_3_ for oxidation in an oil bath at 120 °C for 30 min. The system was allowed to cool to room temperature, the remaining liquid was discarded, and 100 mL of double-distilled water (ddH_2_O) was added to the slurry, which was immediately dispersed. The suspension was washed with ddH_2_O and dialyzed (Spectra/Por^®^; Spectrum Labs, Breda, The Netherlands, 12–14 kDa) against 10 mM HNO_3_ for 48 h by changing the dialysis solution every 10–12 h, and finally, the obtained stable suspensions were stored at 4 °C.

### 3.2. Characterisation of IONPs

Transmission Electron Microscopy (TEM) micrographs of IONPs on carbon grids with 200 Copper meshes (Plano GmbH) were taken with a Philips CM12 microscope (Amsterdam, The Netherlands) with a LaB6 source operated at 120 kV accelerating voltage. Images were recorded with a Gatan 1024 × 1024 pixels MultiScan CCD camera (München, Germany). The long and short ferrets of 500 IONPs were measured manually from randomly taken TEM micrographs using the DigitalMicrograph software (Gatan Inc., München, Germany). The equivalent diameters were subsequently calculated as being the hypothetical diameters of spherical nanoparticles with areas equivalent to the rectangular areas obtained from the long and short ferrets. To assess morphology, aberration-corrected high-resolution TEM micrographs of IONP on lacey carbon grids were taken with an FEI Titan Themis 60–300, primarily operated at 80 kV and with a monochromated incident beam to reduce effects of chromatic aberration, recorded on a CMOS-based FEI CETA 4 k × 4 k camera (FEI, Gräfelfing, Germany).

For all samples, the hydrodynamic diameters and the zeta potentials of 1 mL IONPs’ suspension at concentration of 0.05 mg_Fe_·mL^−1^ at pH 4 were measured at room temperature in acrylic cuvettes (Sarstedt, Nümbrecht, Germany) with a Zetasizer Nano ZS (Malvern Instruments, Worcestershire, UK). The reported values were the average of 3 × 12 measurements. For all samples the iron concentration was determined by ICP-EOS. For this purpose 80 µL of as-synthesized IONPs was mixed with 920 µL of 6 M HCl. After three days, 500 µL of this solution was diluted in 2.5 mL H_2_O and measured. ICP-EOS was performed with ICP-EOS 9000 (Shimadzu, Duisburg, Germany). The Brunauer–Emmett–Teller (BET) specific surface areas *SSA* (m^2^·g^−1^) were determined from N_2_ adsorption isotherms (Gemini 2375, Micromeritics, Verneuil-en-Halatte, France).

Fourier transform IR (FTIR) spectra of IONPs powders were obtained with the Perkin Elmer Spectrum One spectrometer (series: 69288, Perkin Elmer, Schwerzenbach, Switzerland) in ATR mode. Transmittance from 4600 to 400 cm^−1^ was given as the average of measured 8 scans for each curve with a resolution of 4.00 cm^−1^. X-ray Photoelectron Spectroscopy (XPS) measurements were carried out using a PHI VersaProbe II scanning XPS microprobe (Physical Instruments AG, Meylan, France). Analysis was performed using a monochromatic Al Kα X-ray source of 24.8 W power with a beam size of 100 µm. The spherical capacitor analyzer was set at 45° takeoff angle with respect to the sample surface. The pass energy was 46.95 eV yielding a full width at half maximum of 0.91 eV for the Ag 3d 5/2 peak. Curve fitting was performed using the PHI Multipak software (Blue Scientific, Cambridge, UK).

Hyperthermia measurements at frequencies *f* = 229, 248, 264, 314, 352, 440, and 575 kHz and field intensity of 30 mT were carried out on a commercial equipment (DM100, nB Nanoscale Biomagnetics, Zaragoza, Spain). Samples 5 and 6 were measured as prepared, and the other samples after 1/3 dilution, so the γ-Fe_2_O_3_ concentration in the measured suspensions was similar and around 6 g/L in all the heat treated samples. Sample 4 was also measured after gelification in a 22 wt. % gelatin medium to cancel Brownian contribution to the *SAR*, and in a liquid suspension after a 1/2 dilution to observe the effect of the γ-Fe_2_O_3_ concentration on *SAR*. For *SAR* measurements at a frequency of about 100 kHz, and several field intensities ranging from 2 to 42 mT was used homemade equipment. In short, *SAR* measurements were performed with a signal generator (input signal of 7.2 Vpp) connected to an HAS 4014 linear amplifier. The output signal was driven by a matching transformer of ratio *N*_1_/*N*_2_ = 11:3. The secondary load was provided by a RLC tank circuit where *R* = 1 Ω at a resonant frequency of 97.771 kHz: *C* = 20 nF for *f* = 100 kHz and *C* = 5 nF for *f* = 200 kHz. The inductance was provided by a magnetic circuit with MnZn ferrites and a gap of 13 mm. One of the ferrite tips had 10 turns wired to sense the magnetic flux going out of the tip and crossing the gap. The secondary current was measured with a Rogowsky current probe (Power Electronic Measurements Ltd, Nottingham, UK). The magnetic field constant and the maximum secondary current amplitude were 1.348 mT/A and 20 A, respectively. The sample was inserted into plastic cuvettes placed in the ferrite gap and a second cuvette containing H_2_O was used as a reference to measure the heat produced by the ferrite nucleus. The temperatures of the sample and of the reference cuvette were measured with a GaAs temperature sensor (Neoptix Reflex, Ville de Québec, QC, Canada) immersed in the sample and connected to T1 optical fibers with temperature accuracy of ±0.2 K (acquisition rate of 1 Hz). When the temperatures of both the reference and the sample were stable, the temperatures of both probes were recorded during successive periods of time: (i) 30 s with the field off, (ii) 120 s with the field on and (iii) 420 s with the field off. Three runs were performed for each sample. The *SAR* (W·g_Fe2O3_^-1^) was subsequently calculated by the above given Equation (2). The *SAR* values with respect to the mass of γ-Fe_2_O_3_ in the sample were obtained from the derivate of a second order equation fitted to the *T*(*t*) curves. All the *SAR* measurements were repeated at least twice on minimum one device.

## 4. Conclusions

We have studied the structure and the properties of IONPs in aqueous synthesis without ligands with a mild HT treatment as a function of the duration of this treatment. The obtained results revealed the HT durations at which coalescence, recrystallization and the change in the growth mechanism from the solely colloidally driven into also magnetically driven one occurs. The morphology, structure and crystallinity, and thus properties of IONPs were controlled by: (1) the aqueous environment which provides a driving force for coalescence and for the exposure of low energy surfaces through interfacial energy minimization; (2) the absence of capping agents which creates this high-energy interface and allows for surface recrystallization; and (3) the low temperature treatment which permits crystal structure ordering. As a result, the IONPs with favorable multiple properties are obtained at the HT duration when monocrystalline SPs are recrystallized into rectangular shape with nearly defect-free surfaces and diameters, which correspond to the superpara-ferrimagnetic transition. This approach in the challenging control over properties of IONPs can be applied, as well, on the aqueous synthesis (without capping agent(s)) of other compounds as long as PPs are formed prior to the HT treatment. In this way, we have obtained γ-Fe_2_O_3_ IONPs with *SAR* values of up to 1225.1 W·g_Fe_^−1^ over a range of field parameters, but also with a relaxivity *r*_2_ of up to 1189 mM^−1^·s^−1^ and a *r*_2_/*r*_1_ ratio of up to 195.

## Figures and Tables

**Table 1:** Equivalent particle size or TEM diameter (*d*_T_), hydrodynamic diameter (*d*_H_) in water from number-weighted distribution, crystalline diameter (*d*_C_) (the average (*d*_Ca_) and obtained from the narrowest line (404) (*d*_C404_)), lattice parameter (*a*), *ζ*-potential at pH 4 and specific surface area (*SSA*) (measured by BET) of IONPs. Data are given as mean ± standard deviation. Standard deviation for *d*_C_ and *SSA* is ~10%.

Sample Name	*d*_T_ (nm)	*d*_H_ (nm)	*d*_C_ (nm)		*ζ*-Potential at pH 4 (mV)	*a* (Å)	*SSA* (m^2^/g)
			*d*_Ca_	*d*_C404_			
1	8.0 ± 1.9	16.1 ± 4.5	7.6	8.2	55.6 ± 0.4	8.342(9)	170.33
2	14.7 ± 5.0	26.9 ± 8.5	14.6	16.6	47.4 ± 2.2	8.3468(29)	91.92
3	15.6 ± 4.7	29.5 ± 8.5	15.9	18.0	47.9 ± 2.3	8.3505(26)	91.92
4	19.0 ± 5.7	25.8 ± 7.8	15.1	16.8	46.3 ± 1.4	8.3504(25)	80.74
5	17.4 ± 4.7	35.1 ± 10.6	19.5	21.8	49.3 ± 2.4	8.3395(43)	77.53
6	21.5 ± 6.3	30.2 ± 9.1	20.3	22.4	48.2 ± 0.6	8.3519(19)	83.13

## Supplementary Materials

The following are available online at http://www.mdpi.com/2079-4991/7/8/225/s1, additional TEM micrographs, the empirical size distributions, XRD patterns, crystallite diameters, FTIR spectra, XPS spectra, *M*(*H*) curves, hysteresis curves at two temperatures, values of the saturation magnetization, coercivity, remanent magnetization and effective anizotropy constant, thickness of a magnetically dead layer, magnetic volume, relative non-magnetic volume, relaxivities, distributions of hydrodynamic diameters, the transversal relaxation rates as a function of the IONPs concentration, the longitudinal relaxivity vs. TEM diameter, heating curves (in liquid and solid, repetitions, at different concentration of IONPs), *SAR* with the growth of IONPs, *ILP* vs. *f*·*H* product, and 3 videos made by HRTEM.
