# Supplementary material for: Tuning Properties of Iron Oxide Nanoparticles in Aqueous Synthesis without Ligands to Improve MRI Relaxivity and SAR
Source: Nanomaterials (Basel). 2017 Aug 18;7(8):225. doi: 10.3390/nano7080225 (PMC5575707; doi:10.3390/nano7080225)
Supplement: Supplementary file 1 [file nanomaterials-07-00225-s001.zip › nanomaterials-215346-proofdone-supp/Supplementary/nanomaterials-215346-proof-Supplementary revised.pdf]

Supplementary Material

Tuning Properties of Iron Oxide Nanoparticles in Aqueous Synthesis without Ligands to Improve MRI Relaxivity and *SAR*

Debora Bonvin [1], Duncan T.L. Alexander [2], Angel Millán [3], Rafael Piñol [3], Beatriz Sanz [4,†], Gerardo F. Goya [4], Abelardo Martínez [5], Jessica A.M. Bastiaansen [6,7], Matthias Stuber [6,7], Kurt J. Schenk [8], Heinrich Hofmann [1] and Marijana Mionić Ebersold [1,6,7,*]

[1] Powder Technology Laboratory, Institute of Materials, Ecole polytechnique fédérale de Lausanne (EPFL), Lausanne 1015, Switzerland; debora.bonvin@epfl.ch (D.B.); heinrich.hofmann@epfl.ch (H.H.)
[2] Interdisciplinary Centre for Electron Microscopy (CIME), Ecole polytechnique fédérale de Lausanne (EPFL), Lausanne 1015, Switzerland; duncan.alexander@epfl.ch (D.T.L.A.)
[3] Instituto de Ciencia de Materiales de Aragón, Universidad de Zaragoza, C/Pedro Cerbuna 10, 50009 Zaragoza, Spain; amillan@unizar.es (A.M.); pinol@unizar.es (R.P.)
[4] Instituto de Nanociencia de Aragón, Universidad de Zaragoza, Mariano Esquillor s/n, 50018 Zaragoza, Spain; beasanz@unizar.es (B.S.); goya@unizar.es (G.G.)
[5] Grupo de Electrónica de Potencia y Microelectrónica, I3A, Universidad de Zaragoza, 50018 Zaragoza, Spain; amiturbe@unizar.es (A.M.)
[6] Department of Radiology, University Hospital (CHUV) and University of Lausanne (UNIL), Lausanne 1011, Switzerland; jbastiaansen.mri@gmail.com (J.A.M.B.); Matthias.Stuber@chuv.ch (M.S.)
[7] Center for Biomedical Imaging (CIBM), Lausanne 1011, Switzerland
[8] CCC-IPSB, Ecole Polytechnique Fédérale de Lausanne (EPFL), Lausanne 1015, Switzerland; kurt.schenk@epfl.ch (K.J.S.)
* Correspondence: marijanamionic@gmail.com; Tel.: +41-76-238-1669
† Current address: nB nanoScale Biomagnetics S.L., Panamá 2, Local 1-50012-Zaragoza, Spain

Supporting Experimental Section

MRI: Imaging parameters for longitudinal relaxation times T_1: TR 15 s, TE 7.1 ms, slice thickness 5 mm, FOV 250 x 150 mm^2, matrix 384 x 310, RF excitation angle 90°, receiver bandwidth of 651 Hz/pixel, TI 23, 50, 100, 20, 500, 10000, 5000, 10000 ms. Imaging parameters for transversal relaxation times T_2: TR 15 s, TE 7.1, 15, 25, 35, 65, 120, 240 ms, slice thickness 5 mm, FOV 250 x 150 mm^2 , matrix 384 x 310, RF excitation angle 90°, receiver bandwidth of 651 Hz/pixel. The MRI signals of each suspension were determined using Image J and signal evolutions were analysed and fitted using Matlab (The MathWorks, Natick, MA, USA). The signal evolution S as function of TI and TE was fitted to derive the T_1 and T_2 of each γ-Fe_2O_3 nanoparticle suspension respectively, and is described as follows:

(1) $$S(TE) = S(0)e^{-\frac{TE}{T_2}} + C,$$

(2) $$S(TI) = S(0)(1 - 2e^{-\frac{TI}{T_1}}).$$

The T_1 and T_2 values as function of their γ-Fe_2O_3 nanoparticles concentration were subsequently fitted to obtain the relaxivities r_1 and r_2 described as:

(3) $$\frac{1}{T_{1,2}} = \frac{1}{T_{1,2[0]}} + r_{1,2}[\gamma - Fe_2O_3].$$

Hyperthermia measurements: Homemade equipment: SAR measurements were performed with a signal generator (input signal of 7.2 Vpp) connected to an HAS 4014 linear amplifier. The output signal was driven by a matching transformer of ratio N_1/N_2 = 11:3. The secondary load was provided by a RLC tank circuit where R = 1 Ω at a resonant frequency of 97.771 kHz: C = 20 nF for f = 100 kHz and C = 5 nF for f = 200 kHz. The inductance was provided by a magnetic circuit with MnZn ferrites and a gap of 13 mm. One of the ferrite tips had 10 turns wired to sense the magnetic flux going out of the tip and crossing the gap. The secondary current was measured with a Rogowsky current probe. The magnetic field constant and the maximum secondary current amplitude were 1.348 mT/A and 20 A, respectively. The sample was inserted into plastic cuvettes placed in the ferrite gap and a second cuvette containing H_2O was used as a reference to measure the heat produced by the ferrite nucleus. The temperatures of the sample and of the reference cuvette were measured with a GaAs temperature sensor (Neoptix Reflex) immersed in the sample and connected to T1 optical fibers with temperature accuracy of ±0.2 K (acquisition rate of 1 Hz). When the temperatures of both the reference and the sample were stable, the temperatures of both probes were recorded during successive periods of time: i) 30 s with the field off, ii) 120 s with the field on and (iii) 420 s with the field off. Three runs were performed for each sample.

Supporting Figures and Tables

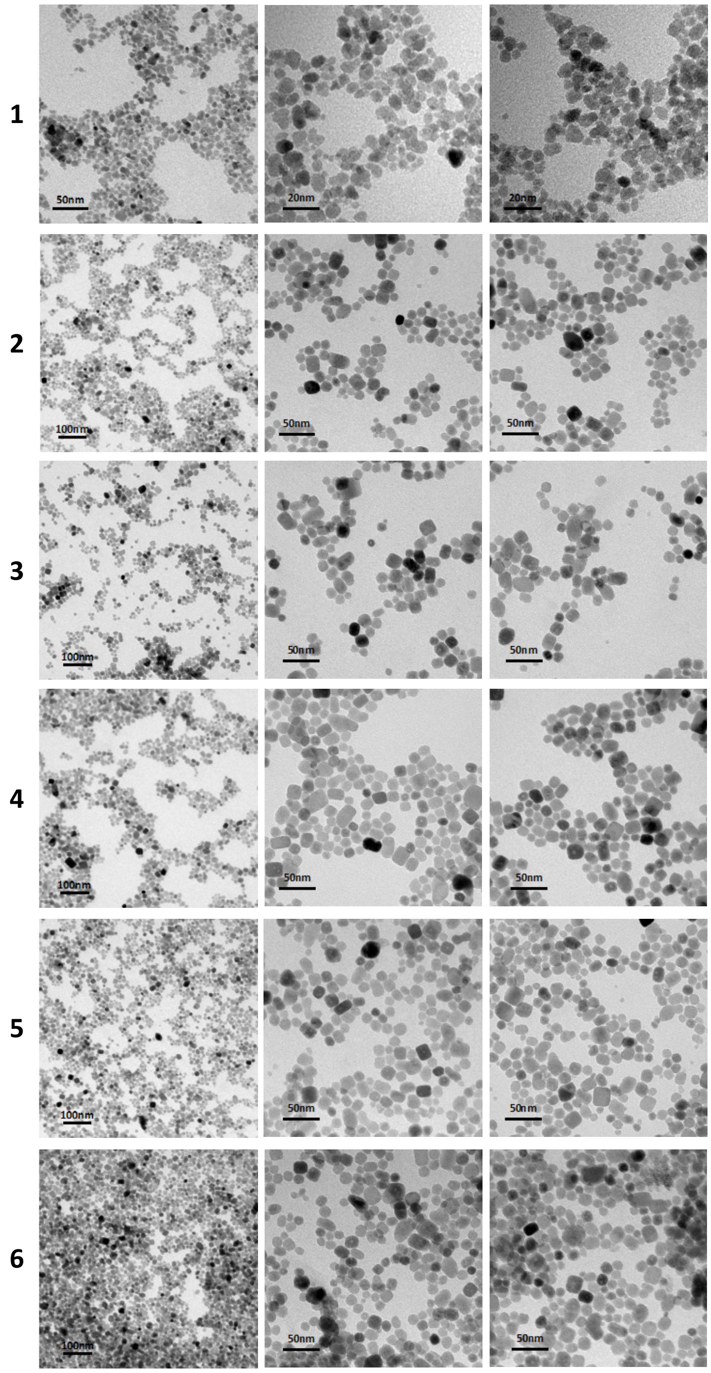

Figure S1. Representative TEM micrographs of the 6 samples of IONPs in addition to the micrographs given in Figure 1(a) (different magnification). Each row displays one sample, starting from the first row showing sample 1, up to the last row showing sample 6.

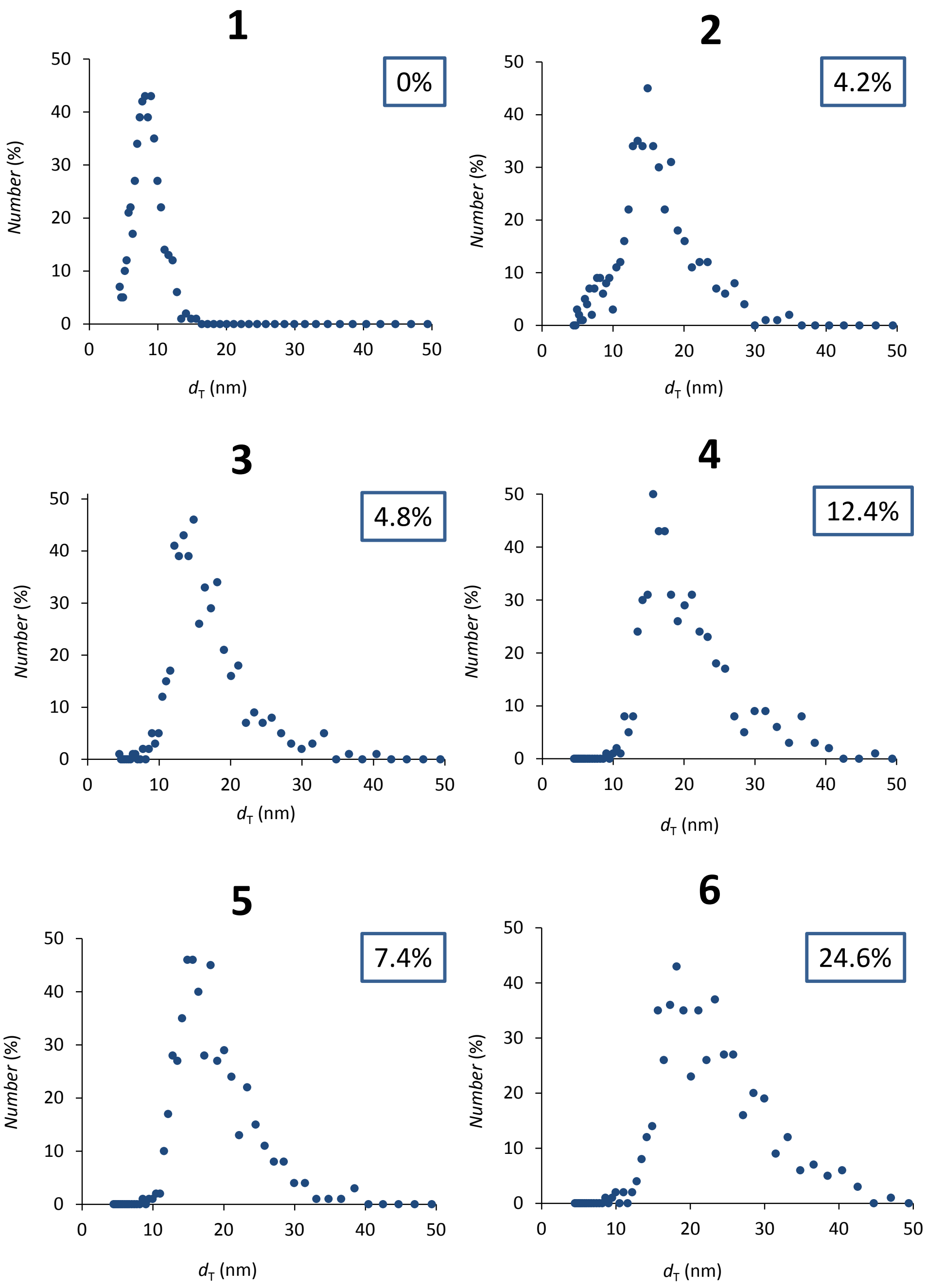

Figure S2. IONPs' empirical TEM diameter (d_T) distribution (blue dots) of 500 IONPs obtained from TEM micrographs (representative micrographs are given in Figure 1(a) and Figure S1) for the 6 IONPs samples. Numbers in blue rectangles in upper right corner of each panel indicate the fraction (in %) of IONPs with d_T equal or larger to 26 nm for corresponding sample.

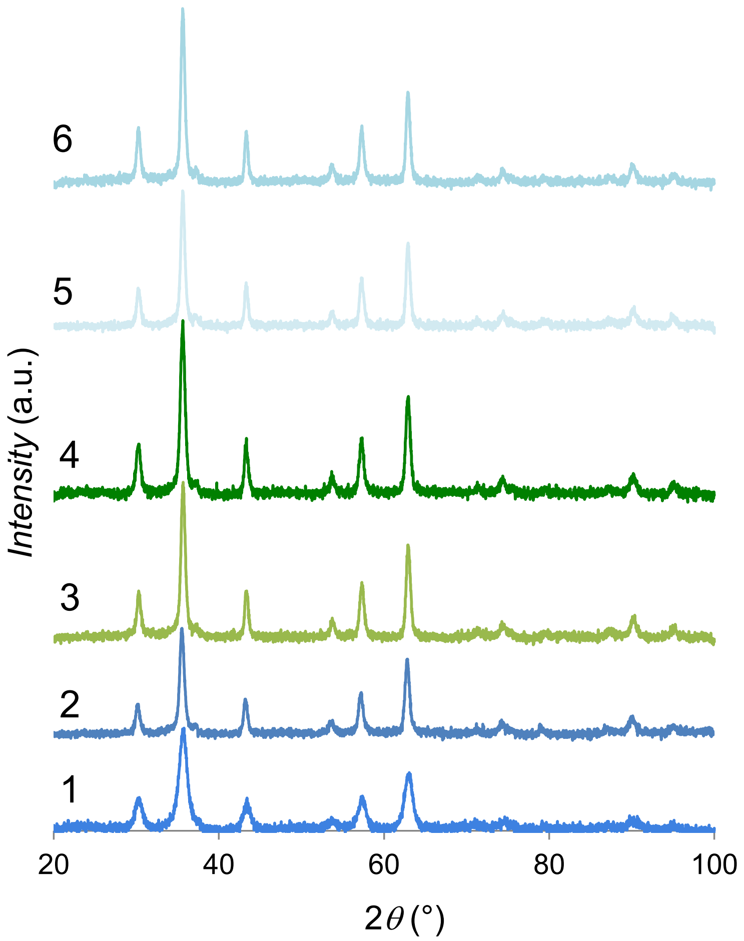

Figure S3. XRD patterns of the 6 IONPs samples with visible peak broadening in sample 1 with the smallest TEM size.

Table S1. Crystallite diameters (d_C) calculated from 8 Bragg's reflections as indicated. The narrowest lines are marked in yellow ((404), (444) and (004)), while the overall crystallite diameters (d_{Ca}) given in blue were calculated as the average of the 8 obtained d_C.

	d_C (nm)					
Bragg's reflection	Sample 1	Sample 2	Sample 3	Sample 4	Sample 5	Sample 6
202	7	12.55	13.7	13.38	17.14	18.14
404	8.23	16.57	17.98	16.76	21.72	22.4
111	7	12.56	13.72	13.4	17.15	18.16
222	6.99	12.55	13.7	13.38	17.12	18.12
444	8.26	16.69	18.12	16.87	21.93	22.62
113	7	12.54	13.7	13.38	17.12	18.12
004	8.22	16.52	17.92	16.71	21.64	22.31
008	8.29	16.82	18.28	17.01	22.17	22.88
d_{Ca} (nm)	7.62375	14.6	15.89	15.11125	19.49875	20.34375

a [Å]	8.342(9)	8.3468(29)	8.3505(26)	8.3504(25)	8.3395(43)	8.3519(19)

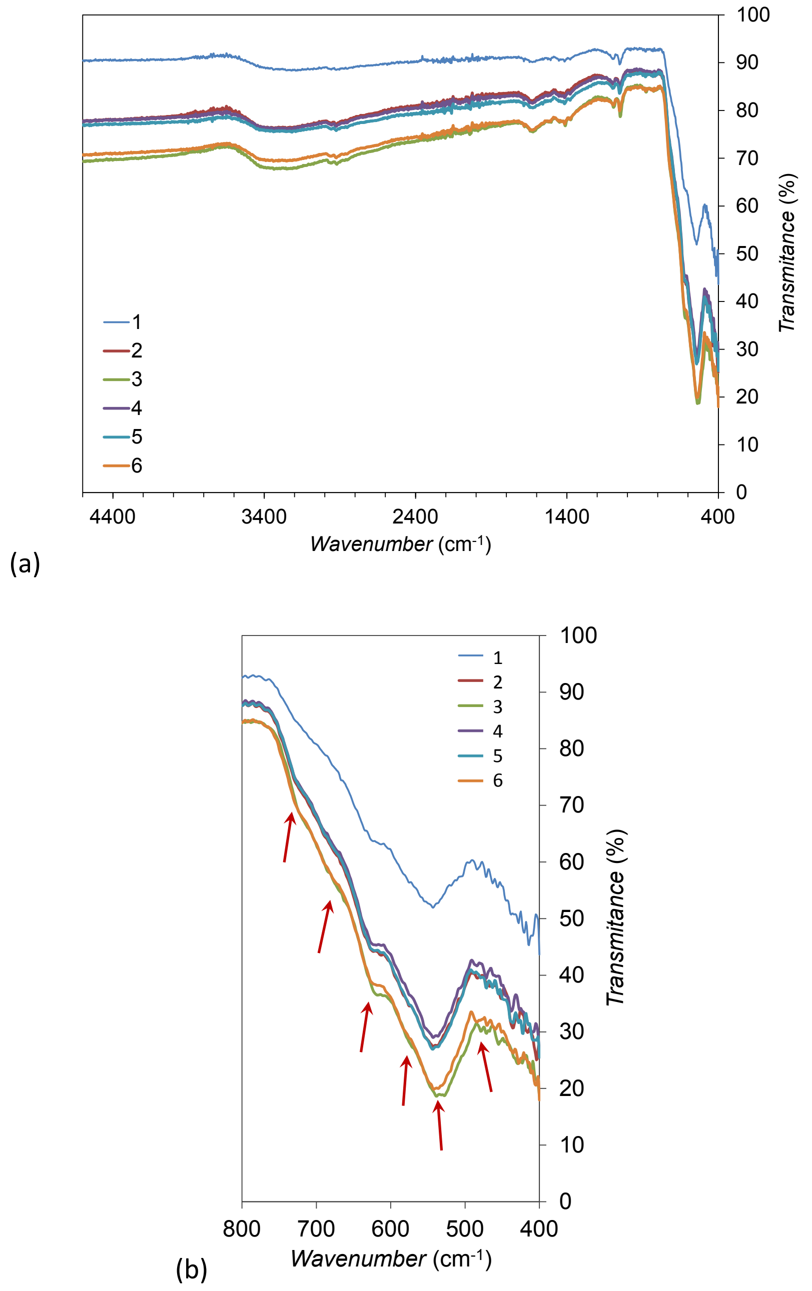

Figure S4. (**a**) Overall and (**b**) magnified region of interest of the FTIR spectra, allowing to differentiate γ-Fe_2O_3 (characteristic peaks are indicated by red arrows in (**b**)) and Fe_3O_4.

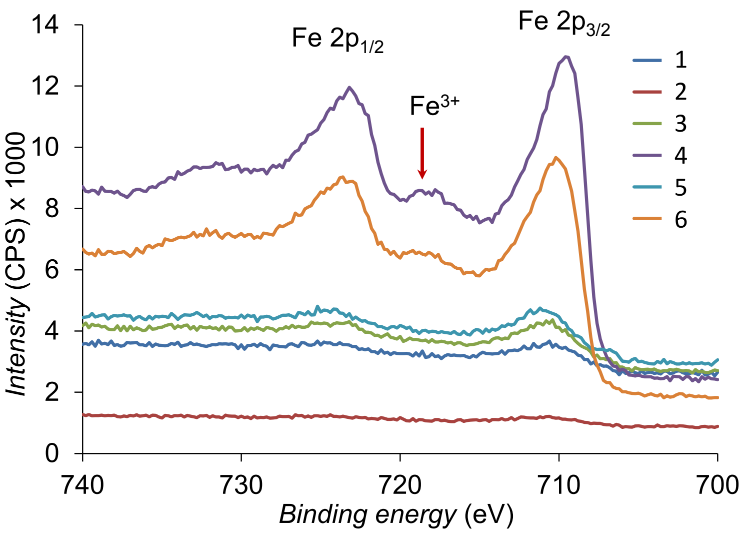

(a)

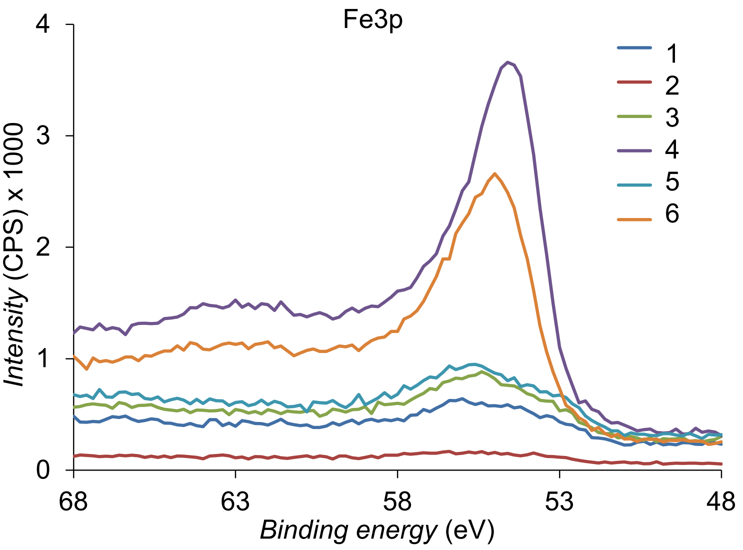

(b)

Figure S5. XPS spectra of IONPs samples at (**a**) the Fe 2p main peak (with the satellite corresponding to the Fe^{3+} peak, which is characteristic for Fe_2O_3) and (**b**) the Fe 3p peak.

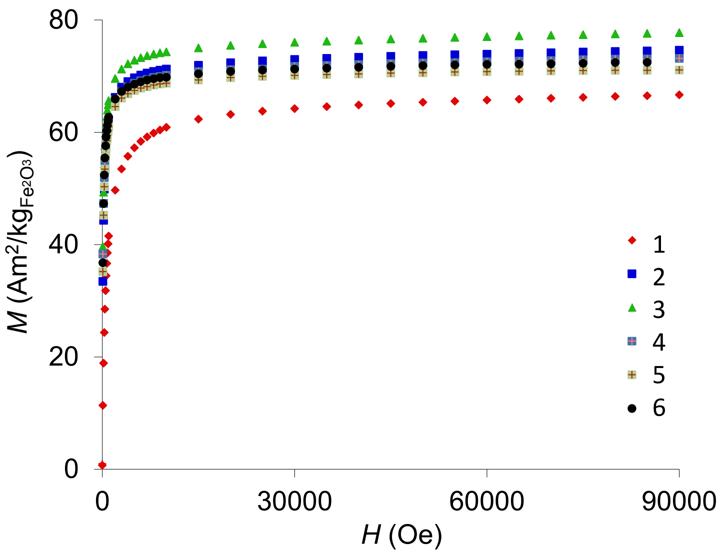

Figure S6. $M(H)$ curves used for the extrapolation of the M_s values from the plateau.

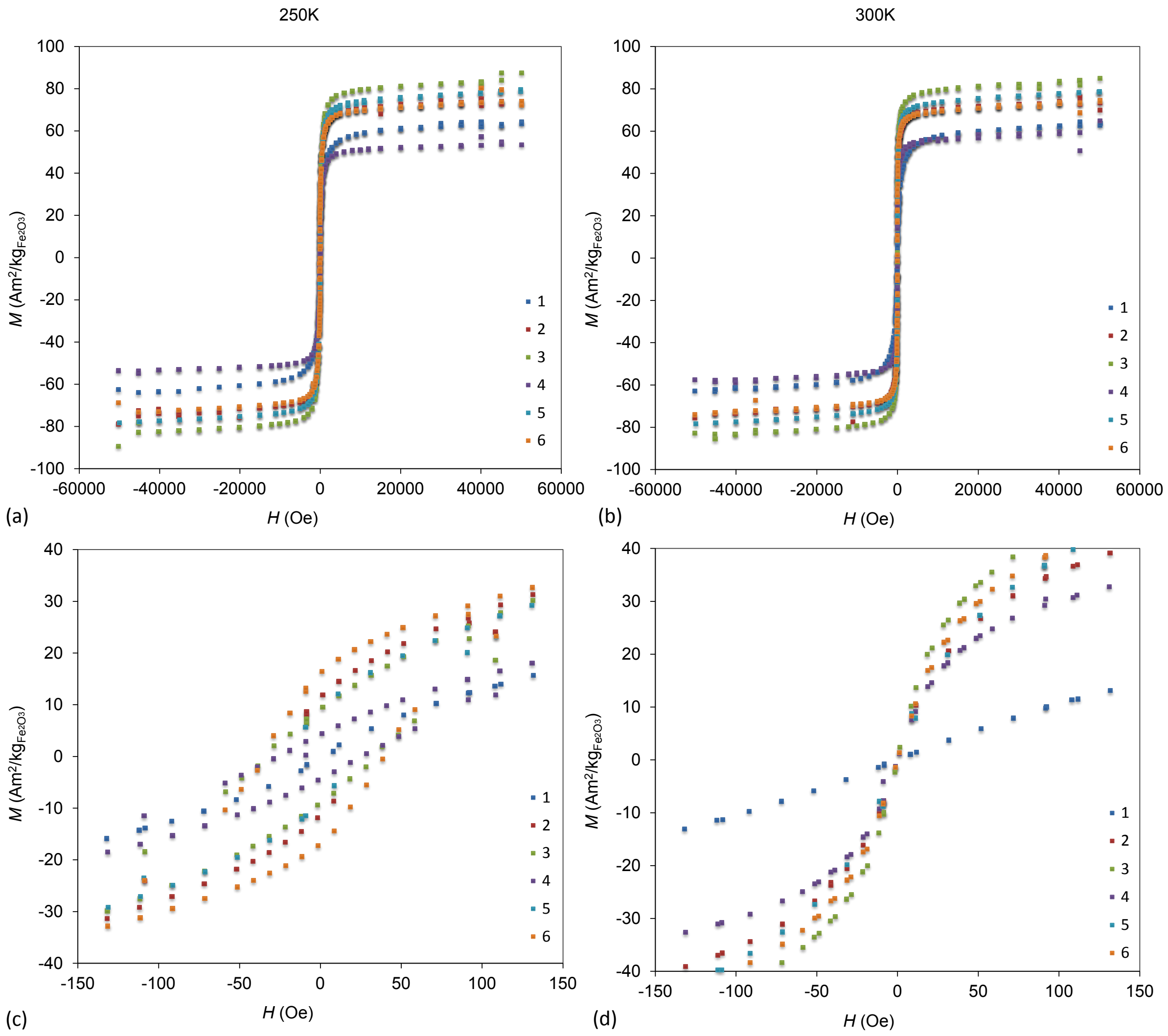

Figure S7. Hysteresis curves at 250 K in the frozen state, and at 300 K in the liquid state (**a**, **b**), as well as the magnified region around zero for both hysteresis curves (**c**, **d**).

Table S2. Saturation magnetization M_s extrapolated from the plateau region in $M(H)$ curves shown in Figure S6, as well as coercivity H_c and remanent magnetization M_r both extrapolated from the hysteresis curves given in Figure S7, and M_r/M_s ratio at 250 K. Effective anisotropy constant (K_{eff}) calculated by the use of H_c and M_s at 250 K in the equation (5) given in the main text.

Sample name	M_s ($A \cdot m^2 \cdot kg_{Fe2O3}^{-1}$)	H_c (Oe) 250 K	H_c (Oe) 300 K	M_r ($A \cdot m^2 \cdot kg_{Fe2O3}^{-1}$) 250 K	M_r ($A \cdot m^2 \cdot kg_{Fe2O3}^{-1}$) 300 K	$M_{r(250\ K)}/M_{s(250\ K)}$	K_{eff} ($J \cdot m^{-3}$)
1	65.5	2.5	0.4	0.0	0.0	0.00	383’282.2
2	74.0	33.6	0.0	11.3	0.0	0.15	57’086.2
3	77.2	34.1	0.2	9.1	0.0	0.11	46’594.8
4	71.1	25.8	-0.2	4.3	0.0	0.08	27’688.1
5	69.8	28.2	-0.3	8.6	0.0	0.11	37’076.0
6	72.0	36.5	-0.1	16.3	0.0	0.23	20’507.5

Table S3. TEM diameter (d_T), volume of IONP approximated as a sphere with the average d_T (V), thickness of a magnetically dead layer (t), magnetic volume (V_m) of IONP approximated as a sphere with the average d_T reduced for two times value of t, relative V_m of an average IONP with respect to its V, and relative non-magnetic volume (V_{nm}) of an average IONP with respect to its V.

Sample	d_T (nm)	V (nm^3)	t (Å)	V_m (nm^3)	V_m/V (%)	V_{nm}/V (%)
1	8.0	267.9	2.3	225.1	84.0	16.0
2	14.7	1’662.4	1.3	1’577.9	94.9	5.1
3	15.6	1’986.8	0.3	1’967.4	99.0	1.0
4	19.0	3’589.5	2.9	3’273.7	91.2	8.8
5	17.4	2’756.9	3.2	2’468.4	89.5	10.5
6	21.5	5’201.1	2.8	4’803.4	92.4	7.6

Table S4. Relaxivities r_2 and r_1 with standard deviations in units $mM_{Fe2O3}^{-1} \cdot s^{-1}$, and the relaxivity ratio r_2/r_1 measured at 3 T at room temperature.

Sample	Relaxivity r_1 ($mM_{Fe2O3}^{-1} \cdot s^{-1}$)	Relaxivity r_2 ($mM_{Fe2O3}^{-1} \cdot s^{-1}$)	r_2/r_1 (-)
1	7.753 ± 0.097	645.1 ± 53.8	83.2
2	5.930 ± 0.046	964.8 ± 30.9	162.7
3	6.176 ± 0.128	987.1 ± 24.1	159.8
4	5.509 ± 0.104	814.2 ± 36.1	147.8
5	5.458 ± 0.061	971.6 ± 22.3	178.0
6	6.094 ± 0.116	1189 ± 48.6	195.1

Table S5. Relaxivities r_2 and r_1 with standard deviations in units ($s^{-1} \cdot \mu g_{Fe}^{-1} \cdot ml$), as well as the relaxivity ratio r_2/r_1 for our 6 IONPs' samples and for the former commercial contrast agent Resovist measured at 3T at room temperature.

Sample	Relaxivity r_1 ($s^{-1} \cdot \mu g_{Fe}^{-1} \cdot ml$)	Relaxivity r_2 ($s^{-1} \cdot \mu g_{Fe}^{-1} \cdot ml$)	r_2/r_1 (-)
1	0.06942 ± 0.00092	5.773 ± 0.489	83.2
2	0.05310 ± 0.00036	8.639 ± 0.276	162.7
3	0.05530 ± 0.00114	8.840 ± 0.208	159.8
4	0.04933 ± 0.00100	7.290 ± 0.329	147.8
5	0.04888 ± 0.00050	8.700 ± 0.203	178.0
6	0.05458 ± 0.00096	10.650 ± 0.432	195.1
Resovist	0.06999 ± 0.00221	4.291 ± 0.126	61.3

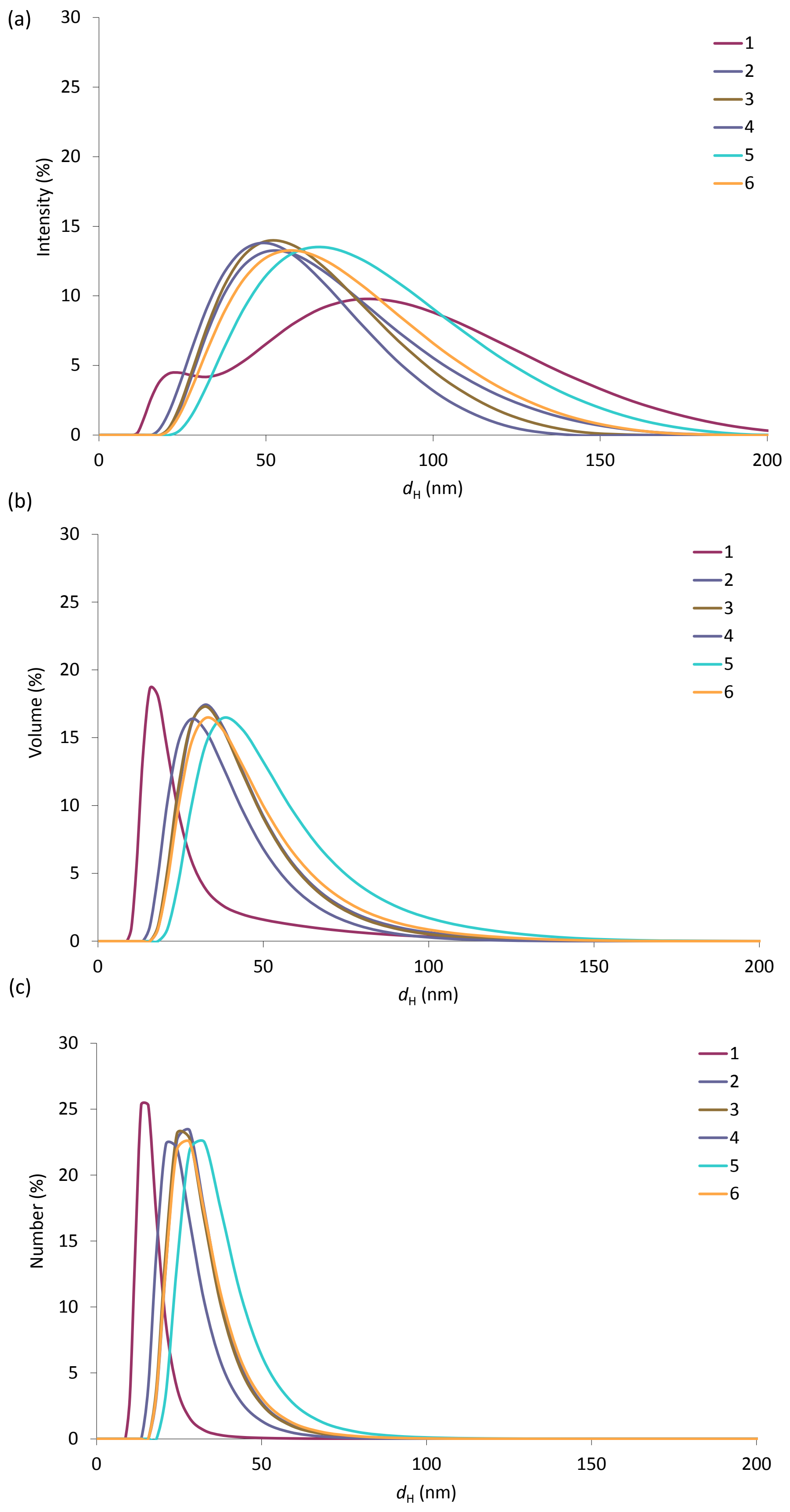

Figure S8. (**a**) The intensity-, (**b**) volume- and (**c**) number-weighted distribution of hydrodynamic diameters (d_H) of IONPs in the 6 samples.

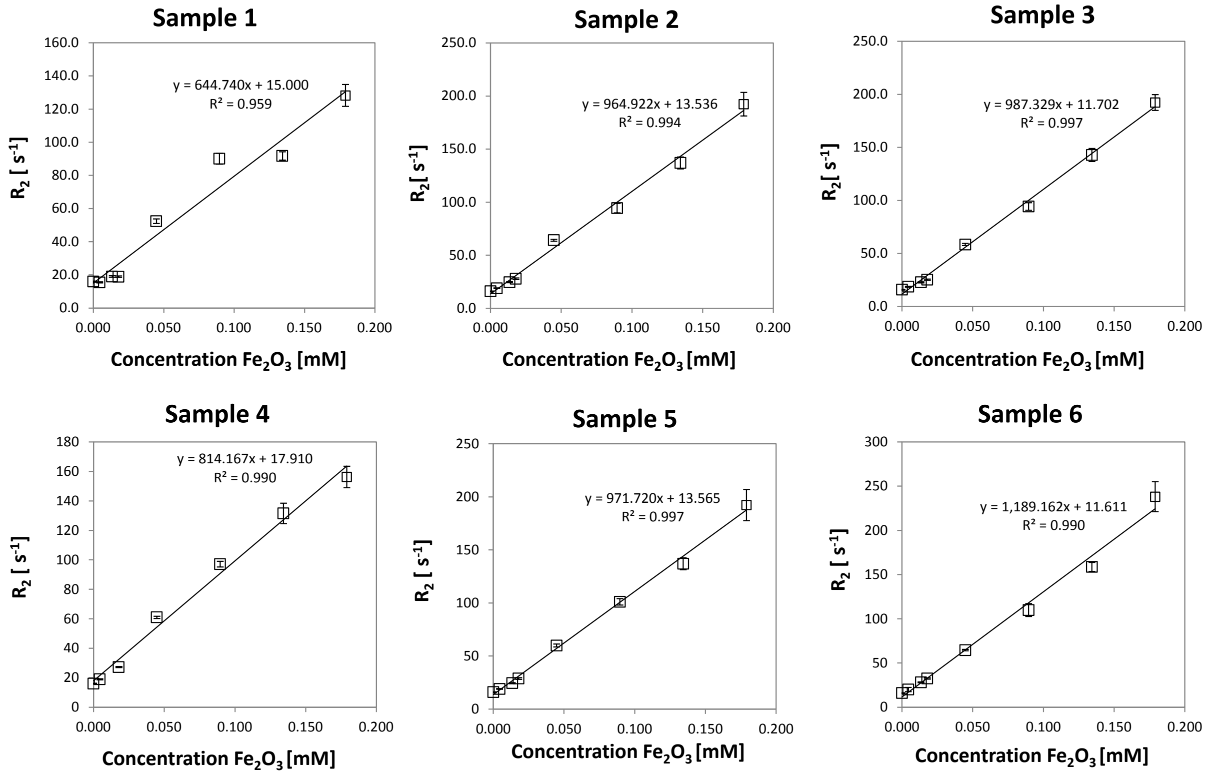

Figure S9. The transversal relaxation rate (*R*2) as a function of the IONPs concentration.

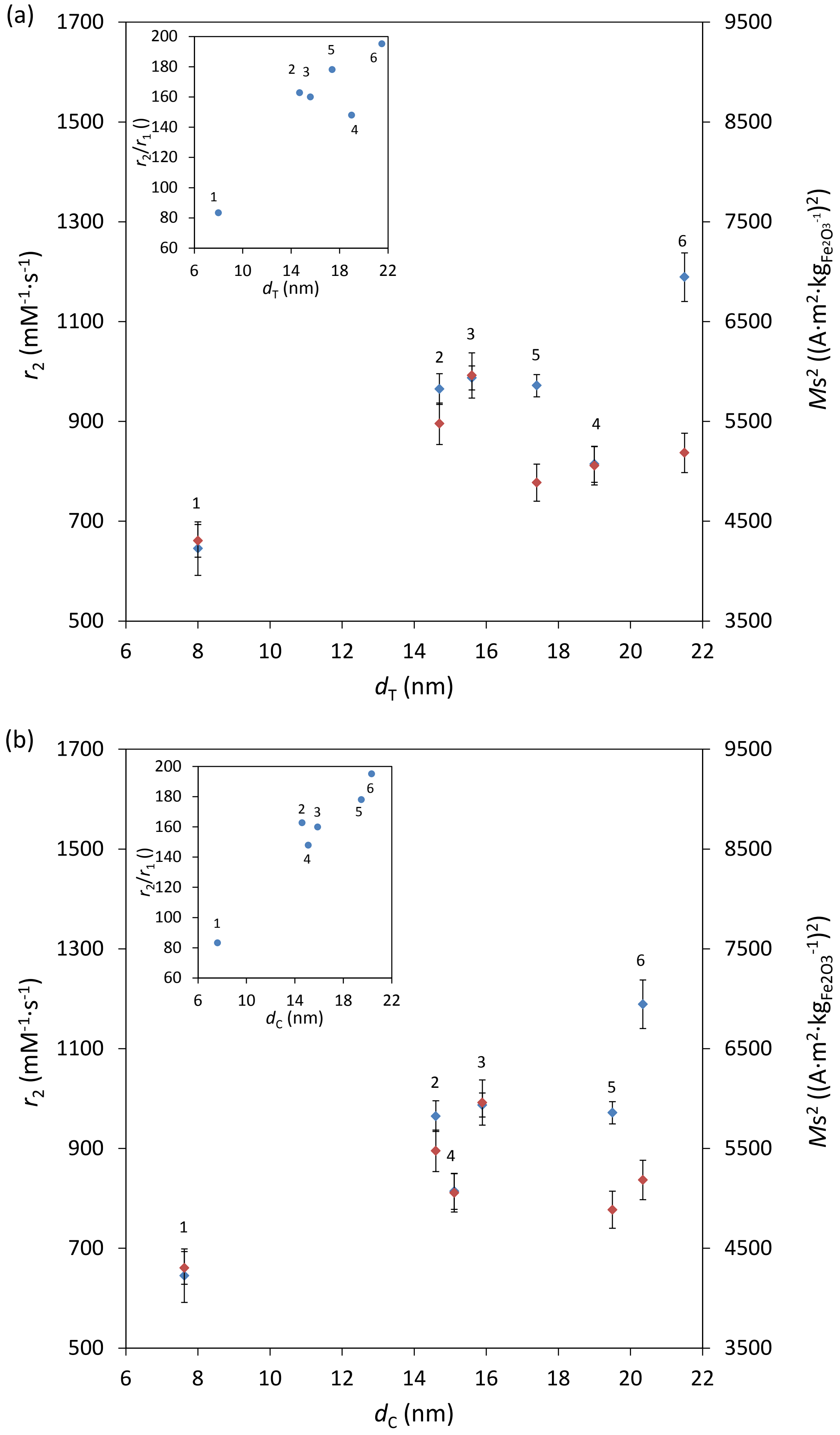

Figure S10. Plot of r_2 and $(M_s)^2$ as a function of d_T (**a**) and d_C (**b**).

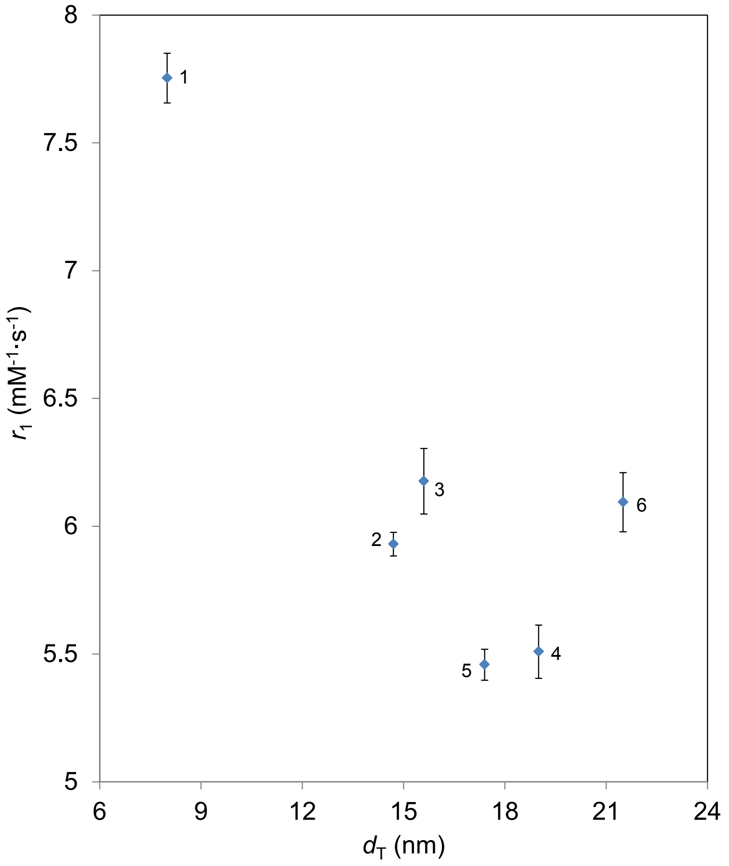

Figure S11.The r_1 values (with indicated standard deviations) as a function of the TEM diameter, d_T.

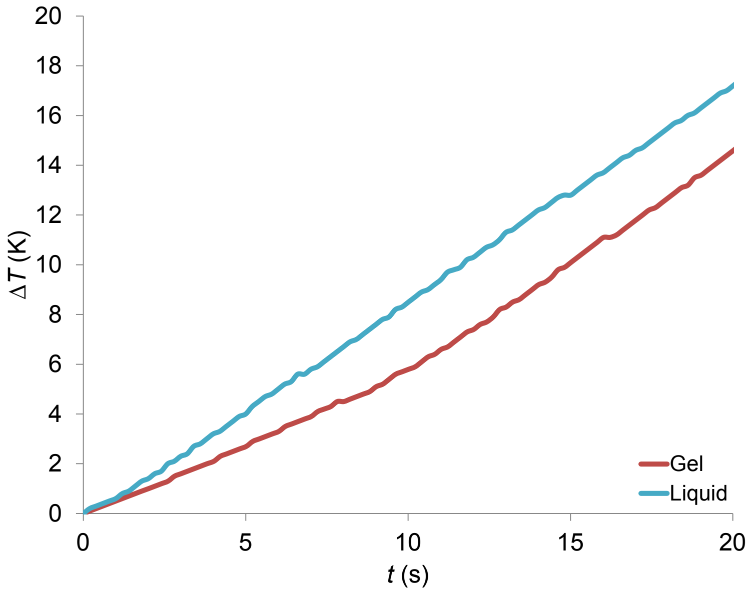

Figure S12. An example of two heating curves for the same sample measured in water and in an agar-gel, showing that *SAR* decreases in agar due to the fixed IONPs and thus due to the loss of the Brownian contribution to the heat dissipation.

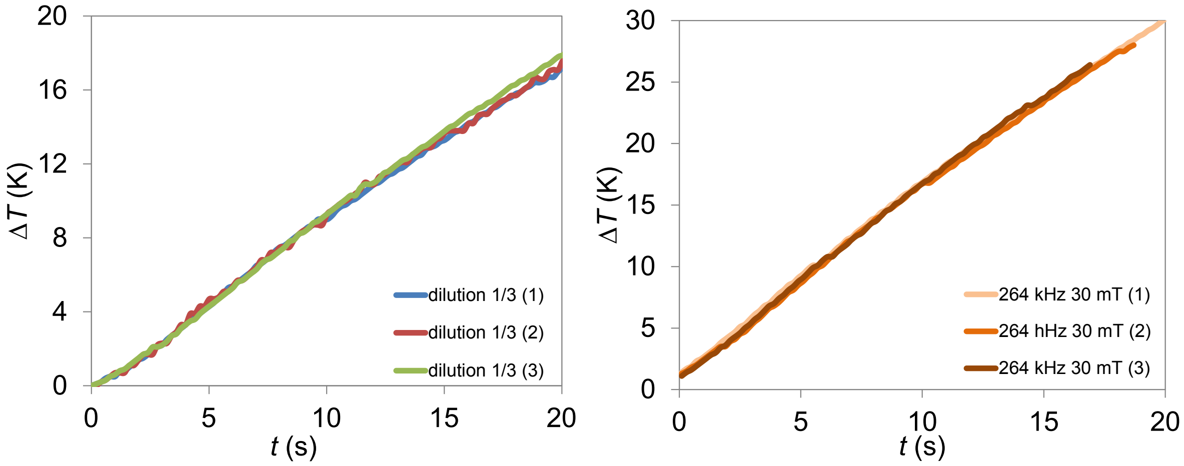

Figure S13. An example of the repeatability of the *SAR* measurements given for two different samples (3 repetition of heating curves per sample).

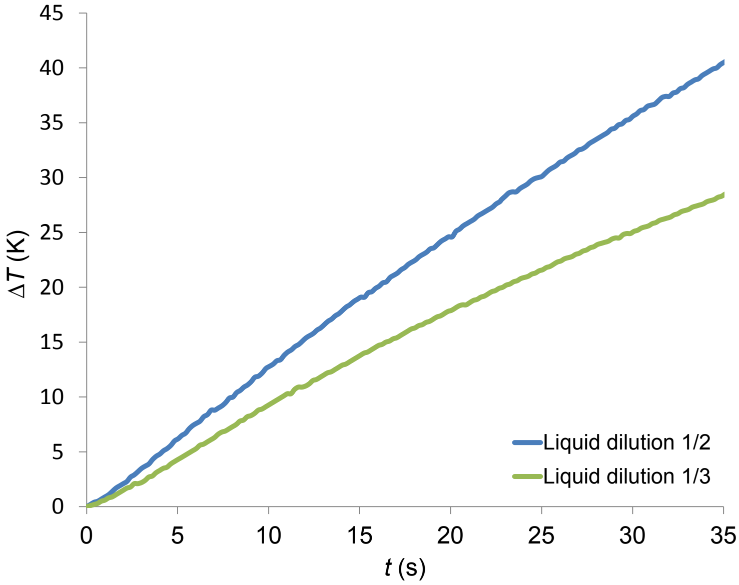

Figure S14. An example of two heating curves of the same sample at different IONPs' concentrations, showing that the slope of the curve changes proportionally to the concentration, resulting in the same *SAR* in both cases.

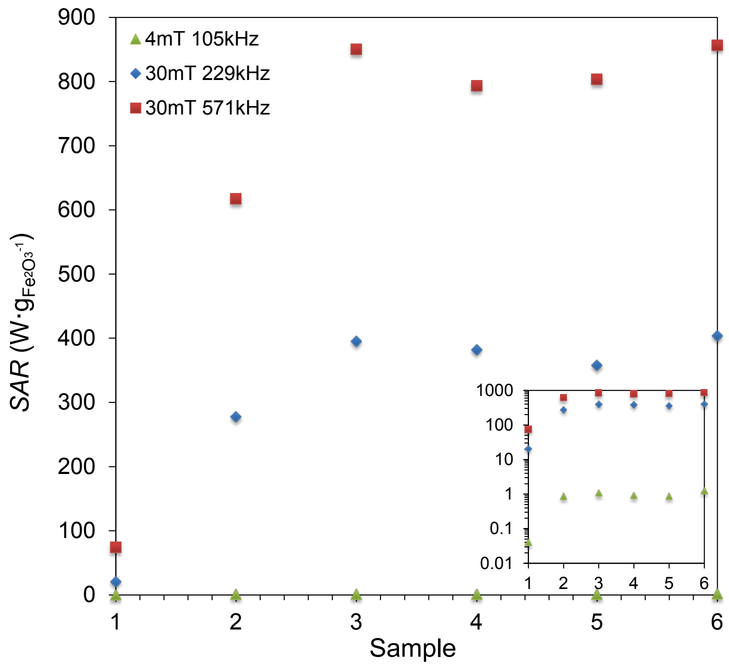

Figure S15. *SAR* versus the sample's number (with the insert showing the same graph with logarithmic y-axe) showing the evolution of *SAR* with the growth of IONPs.

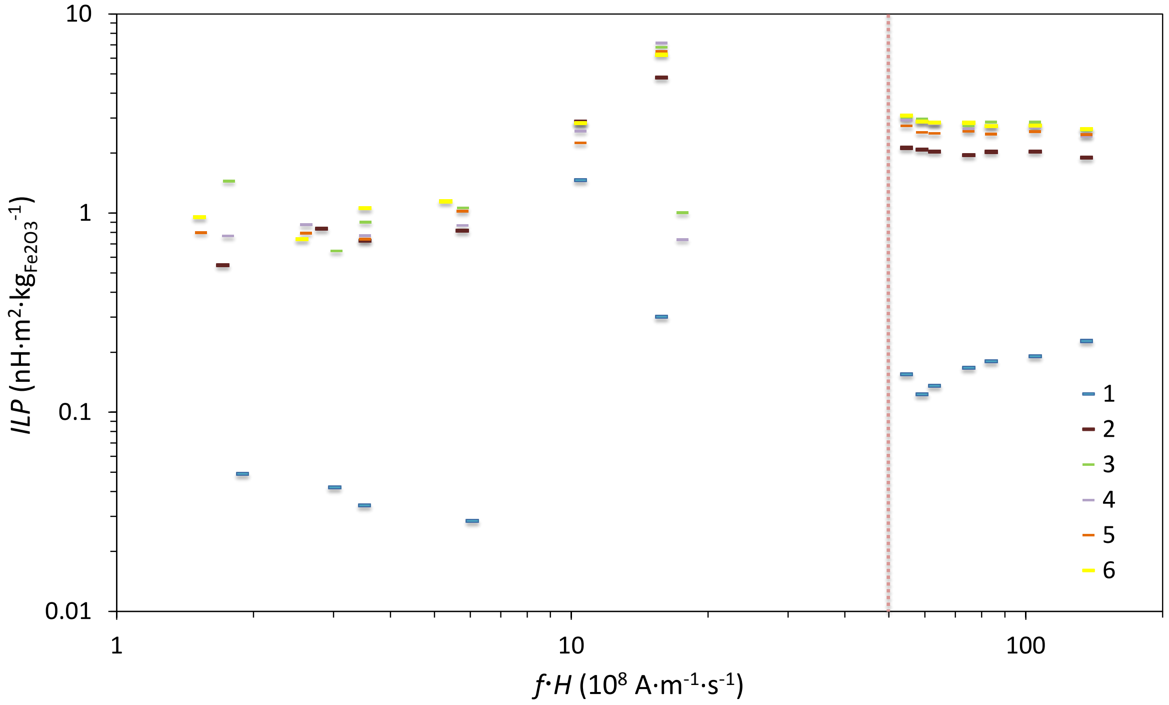

Figure S16. The *ILP* values given as a function of the *f·H* product for all conditions as in Figure 6 (b), including boundary condition for clinical applications (vertical dashed line for the limits of the *f·H* product).
